# Transcriptome-based identification of new anti-anti-inflammatory and vasodilating properties of the n-3 fatty acid docosahexaenoic acid in vascular endothelial cell under proinflammatory conditions

**DOI:** 10.1371/journal.pone.0129652

**Published:** 2015-06-26

**Authors:** Marika Massaro, Rosanna Martinelli, Valentina Gatta, Egeria Scoditti, Mariangela Pellegrino, Maria Annunziata Carluccio, Nadia Calabriso, Tonia Buonomo, Liborio Stuppia, Carlo Storelli, Raffaele De Caterina

**Affiliations:** 1 National Research Council (CNR), Institute of Clinical Physiology, Lecce, Italy; 2 CEINGE Biotecnologie Avanzate, Naples, Italy; 3 Department of Medicine and Surgery of Salerno University, Salerno, Italy; 4 “Gabriele d’Annunzio” University and Center of Excellence on Aging, Chieti, Italy; 5 Department of Biological and Environmental Science and Technology (Disteba), University of Salento, Lecce, Italy; 6 Fondazione Toscana “Gabriele Monasterio”, Pisa, Italy; University Hospital Medical Centre, GERMANY

## Abstract

**Scope:**

High intakes of n-3 fatty acids exert anti-inflammatory effects and cardiovascular protection, but the underlying molecular basis is incompletely defined. By genome-wide analysis we searched for novel effects of docosahexaenoic acid (DHA) on gene expression and pathways in human vascular endothelium under pro-inflammatory conditions.

**Methods and Results:**

Human umbilical vein endothelial cells were treated with DHA and then stimulated with interleukin(IL)-1β. Total RNA was extracted, and gene expression examined by DNA microarray. DHA alone altered the expression of 188 genes, decreasing 92 and increasing 96. IL-1β changed the expression of 2031 genes, decreasing 997 and increasing 1034. Treatment with DHA before stimulation significantly affected the expression of 116 IL-1β-deregulated genes, counter-regulating the expression of 55 genes among those decreased and of 61 among those increased. Functional and network analyses identified immunological, inflammatory and metabolic pathways as the most affected. Newly identified DHA-regulated genes are involved in stemness, cellular growth, cardiovascular system function and cancer, and included cytochrome p450 4F2(CYP4F2), transforming growth factor(TGF)-β2, Cluster of Differentiation (CD)47, caspase recruitment domain(CARD)11 and phosphodiesterase(PDE)5α.

**Conclusions:**

Endothelial exposure to DHA regulates novel genes and related pathways. Such unbiased identification should increase our understanding of mechanisms by which n-3 fatty acids affect human diseases.

## Introduction

Numerous observational and intervention studies have proven beneficial effects of the n-3 polyunsaturated fatty acids (PUFAs) eicosapentaenoic acid (EPA) and docosahexaenoic acid (DHA) in inflammatory diseases, including atherosclerosis [1], and cancer [2]. The vascular endothelium is altered in the initiation and development of atherosclerosis [3], and in the aberrant angiogenesis [4] occurring in plaque instability, diabetes, and solid cancer [5], and appears to be an important target for such effects [6].

Although several recent studies have demonstrated that n-3 PUFAs may positively affect endothelial dysfunction [7], a comprehensive evaluation of the endothelial genomic effects exerted by n-3 PUFAs is lacking [8]. Microarray analysis, the high-throughput genomic tool that allows the simultaneous comparison of thousands of genes, is a useful tool to investigate pathophysiological mechanisms involved in a variety of human diseases, including atherosclerosis [9], and cancer [10], in a fashion not biased or restricted by a priori hypotheses. Cluster analysis of resulting data on transcriptional profiling also has the potential of revealing distinctive patterns of gene expression, thus providing novel information on the influence of n-3 PUFAs in vascular pathophysiology.

With this background, we here assessed by microarray analysis the global pattern of changes in gene expression occurring in cultured human endothelial cells exposed to DHA under pro-inflammatory conditions.

## Materials and Methods

### Materials

DHA (22:6 n-3 all *cis*) was obtained as 99% pure sodium salts from Nu-Chek (Elysian, MN, USA). All other reagents were purchased from Sigma-Aldrich (St. Louis, MO, USA).

For further details, see S1 File.

### Cell isolation and culture

Human umbilical vein endothelial cells (HUVECs) were harvested and maintained as described previously [11]. In particular, human cells were obtained from discarded umbilical veins, and treated anonymously, conforming with the principles outlined in the Declaration of Helsinki. The authors did not collect the tissues themselves, and cells were anonymized before use by the authors. As such, approval from the University Ethics Review Board was not necessary (see ref. [12] for previous use of such internal rule). For further details, see S1 File.

### Experimental design and RNA extraction

HUVECs were preincubated with 50 μmol/L DHA for 48 h, followed by stimulation with 5 ng/mL interleukin (IL)-1β, for additional 0–3 h, after which time cells were collected and total RNA extracted using the Qiagen RNeasy kit (Qiagen, Milan, Italy) according to manufacturer’s instructions. Concentration and purity of RNA was determined by NanoDrop ND-1000 UV-Vis Spectrophotometry (NanoDrop Technologies, Wilmington, DE, USA). No variation in the total RNA yield was observed under the different experimental conditions tested (data not shown).

### Microarray analysis

For microarray analysis, RNAs were labeled and hybridization performed using the Gene Expression Hybridization kit (Agilent Technologies Inc., Santa Clara, CA, USA) following the manufacturer’s instructions. Gene expression profiles were generated using the 4x44K glass slide Whole Human Genome Oligo Microarray G4112A (Agilent Technologies). Each array assessed total RNAs from treated endothelial cells (DHA, IL-1 or DHA + IL-1), with RNA obtained from control endothelial cells (untreated endothelial cells). Raw data were processed by the GeneSpring 10 software (Agilent Technologies), as previously described [13]. Microarray data were made public by reporting them in the Gene Expression Omnibus (GEO) public database. The accession number is: GSE57825. For further details, see S1 File.

### Network identification and canonical pathway analysis

Lists of genes significantly regulated by DHA and/or IL-1β were analyzed by the Ingenuity Pathways Analysis (IPA) software (Ingenuity Systems, Redwood City, CA, USA). For further details, see the S1 File.

### Real-time PCR analysis

To validate microarray data, quantitative real-time PCR (qRT-PCR) was performed on the same samples used for microarrays experiments and on additional samples obtained under the same experimental conditions. Primer designs were based on the entire coding region for each gene **(**see Table A in S2 File). For further details, see S1 File.

### Protein extraction and Western blotting

In order to verify whether the identified changes in gene expression translate in modulation of the corresponding protein HUVEC were preincubated with 50 μmol/L DHA for 48 h, followed by stimulation with 5 ng/mL IL-1β for additional 0–24 h. After this time cells were lysed and processed by sodium dodecyl sulfate-polyacrylamide gel (SDS-PAGE). For further details, see S1 File.

### Knockdown of CD47 and CARD11 by small interfering RNA(siRNA)

Gene knockdown experiments were performed by transient transfection, exposing HUVECs to a pool of pre-designed siRNA (Qiagen) against CD47 (FlexiTube siRNA: Hs_CD47 _6, 7, 8), CARD11 (FlexiTube siRNA: Hs_CAR11 _1, 4, 7) or to a scrambled sequences (AllStar Negative Control siRNA, 1027281, Qiagen) using the DharmaFECT Transfection Reagent (Dharmacon, CO, USA) according to the manufacturer’s protocol. For further details, see S1 File **.**

### Statistical analysis and comparisons

Raw data were processed with the GeneSpring 10 software (Agilent Technologies), and differentially expressed RNA were identified using the Benjamini and Hochberg False Discovery Rate (FDR), with a P value for significance at set at 0.05. Student’s t-test for paired observations was used for comparisons of qRT-PCR results. Data are expressed as fold change (FC). Down-regulated genes were defined as FC <1.5, and up-regulated genes as FC >1.5. P values of 0.05 were accepted to indicate statistically significant differences.

## Results

### Global characterization of gene expression changes

**Fig 1:**
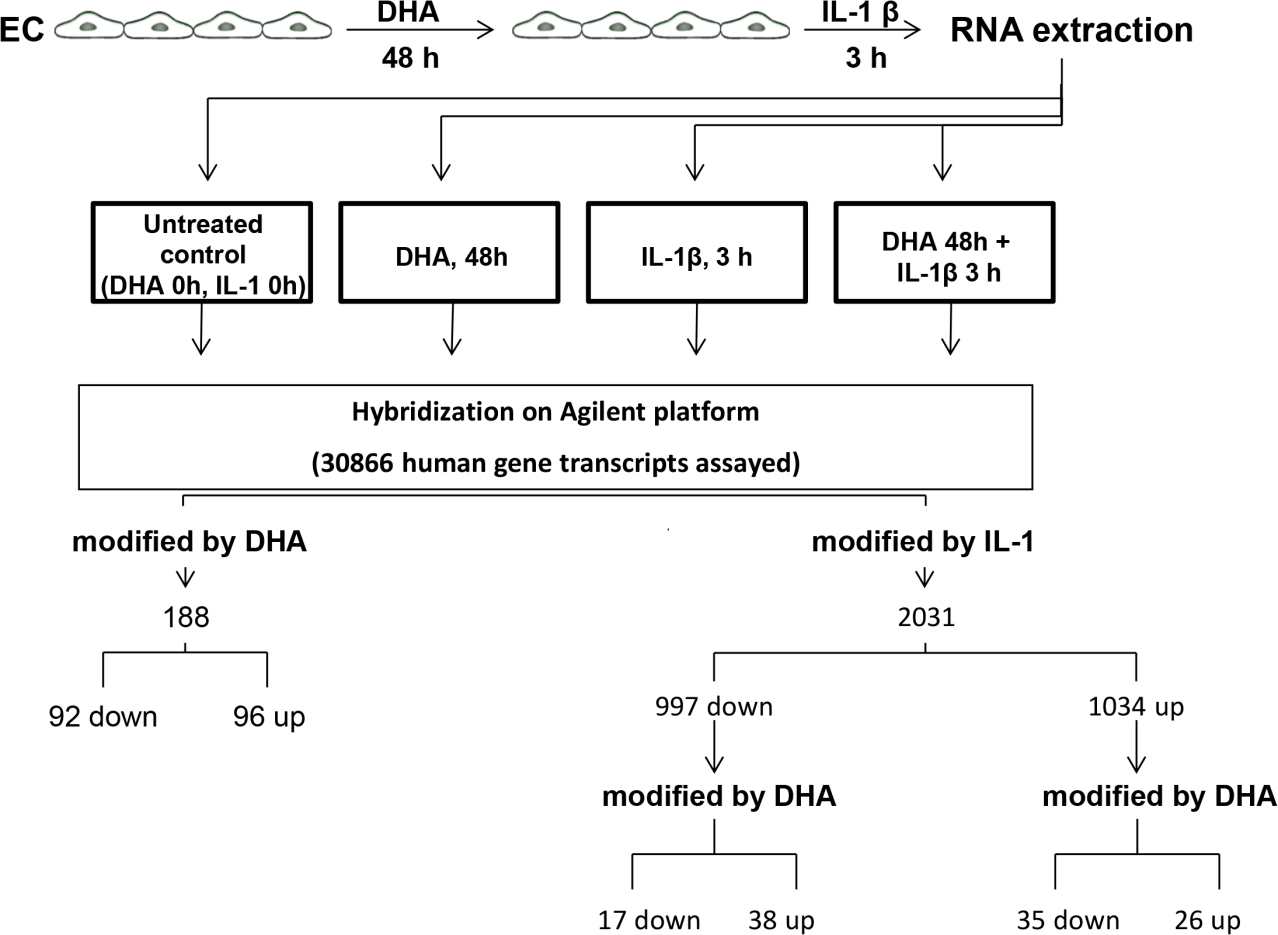
Experimental design and primary conclusions of the study. Abbreviations: EC, endothelial cells; DHA, docosahexaenoic acid; IL-1, interleukin-1 β.

We had previously shown that DHA, starting from 25 μmol/L for 48 h, inhibited endothelial adhesion molecule expression, without endothelial toxicity, in a setting compatible with that of maximal DHA uptake and incorporation into endothelial cell membrane phospholipids [14]. All experiments were therefore performed accordingly. Comparative analysis of DHA-treated cells and untreated control cells identified that less than 1% of interrogated genes are significantly regulated, half of which (96 genes) were increased in expression and the rest (92 genes) were decreased (Fig 1). In contrast, 3 h stimulation with IL-1β 5 ng/mL affected a much higher number of genes, with 6.5% of the assayed gene sequences resulting in significant changes, with increased expression of 1034 genes and decreased expression of 997 other genes (Fig 1). Globally, treatment with DHA before IL-1β stimulation altered the expression of more than 2% of the IL-1β regulated genes (Fig 1).

### Functional categorization of DHA- and IL-1β-regulated genes

#### Effects of DHA on endothelial baseline gene expression

The categorization of regulated genes according to their function showed that DHA per se, compared with control conditions (absence of any treatment), regulated the expression of genes mainly associated with biological processes including cancer, tissue morphology, antigen presentation, cell-to-cell signaling and interaction, and cell-mediated immune response (Table B in S2 File). Although with lower scores, categories of cardiovascular system development and function, as well as cardiovascular disease also resulted significantly affected by DHA (Table C in S2 File). Among these biological processes, the largest sets of genes modulated by DHA were functionally related to hematological neoplasms and mammary tumors (13 and 14 molecules, respectively), blood vessel remodeling, and cardiovascular tissue morphology (5 and 4 molecules, respectively), cell activation (14 molecules), leukocyte movement (10 molecules), regulation of blood pressure and cardiovascular system morphology (7 and 10 molecules, respectively). The most cited gene of cardiovascular interest were transforming growth factor (TGF)-β2, angiopoietin (ANGPT)-1 and CD47, the expression of which was reduced by 34%, 40%, and 35% respectively (with FC of 1.51, 1.67, 1.54, P<0.05% vs control) and cytochrome P 450 (CYP)4F2, increased by 152%, with a FC of 2.52 (P<0.05% vs control).

**Fig 2:**
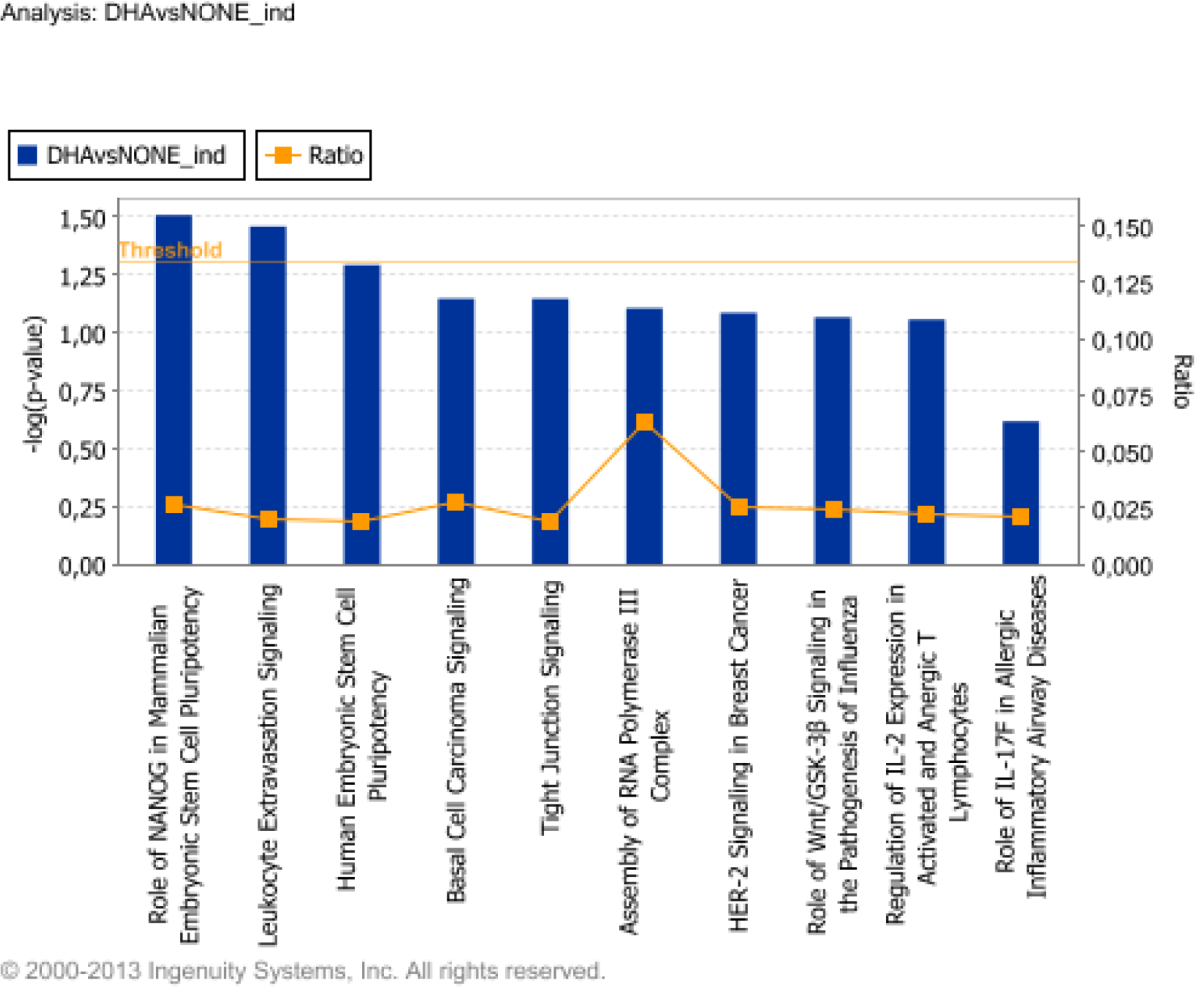
Top ten signaling and metabolic pathways regulated by docosahexaenoic acid (DHA) in resting, unactivated conditions. For the functional categorization of genes the Fisher’s exact test was used to calculate a P value (shown as bars), indicating the probability that each biological function assigned to the network is due to chance alone. The ratio represents the number of differentially expressed genes in a given pathway divided by total number of genes making up that canonical pathway.

IPA identified several interesting pathways associated with DHA treatment (Fig 2). The top canonical pathways, based on their significance (P value), included: role of Nanog in mammalian embryonic stem cell pluripotency, leukocyte extravasation signaling, tight junction signaling, and regulation of interleukin(IL)-2 expression in activated and anergic T lymphocytes. Genes included in each group of the top ten signaling pathway are listed in Table D in S2 File. When IPA was inquired for network analysis, it yielded 11 significant regulatory networks with a score >2. The number one network here ranked (score = 44, focus molecules = 22) was associated with cell movement, hematological system development and function, and immune cell trafficking (S1 Fig). Top functions of the subsequent 4 highly significant networks included cancer, lipid metabolism and cardiovascular disease.

#### Effect of DHA on the expression of IL-1β-regulated genes

Cell stimulation with IL-1β changed the expression of genes mainly associated with cellular development, cell death and survival, the development and function of cardiovascular and hematological systems, and tissue morphology (Table E in S2 File). Among these, most genes modulated by IL-1β appeared to be related to cell differentiation (283 genes), tumor cell proliferation (178 genes), apoptosis (315 genes), and the development of cardiovascular system and blood vessels (168 and 135 genes, respectively). Results of canonical pathway analysis again revealed several pathways as significantly associated with IL-1β stimulation (S2 Fig). The top 10 canonical pathways, ranked by their significance (P value) included: the role of IL-17A in arthritis, the activation of hepatic fibrosis/hepatic stellate cell, the role of protein kinase receptor (PKR) in interferon induction and antiviral response, the role of macrophages, fibroblasts and endothelial cells in rheumatoid arthritis, and the role of IL-17F in allergic inflammatory airway diseases.

To evaluate whether and how DHA affects the endothelial inflammatory response as by IL-1β, we compared the gene expression profile of HUVECs treated with DHA and then stimulated with IL-1β with those of HUVECs only stimulated with IL-1β. Here we selected, among genes regulated by DHA + IL-1β vs IL-1β, only those for which expression values diverged by at least of 1.5 FC from IL-1β (Tables 1 and 2).

**Table 1:** Genes differentially expressed in DHA plus IL-1β versus IL-1β-treated cells.

Probe Name	FC (IL-1 vs None)	Regulation	FC (DHA plus IL-1 vs IL-1)	Regulation	Symbol	Description	Genbank Accession
A_24_P687	1,73	down	1,5742	up	CDH6	Cadherin 6	NM_004932
A_24_P221154	1,30	down	1,6453	up	ABCD3	ATP-binding cassette	NM_002858
A_24_P100130	1,10	down	1,7559	up	BCL2L1	BCL2-like 1	NM_001191
A_23_P120354	1,38	down	1,5307	up	ANKRD57	Ankyrin repeat domain 57	NM_023016
A_24_P98109	1,84	down	1,5377	up	SNX10	Sorting nexin 10	NM_013322
A_24_P7600	1,09	down	1,8849	up	FBXL7	F-box and leucine-rich repeat protein 7	NM_012304
A_23_P67278	1,54	down	1,8525	up	ZNF443	Zinc finger protein 443	NM_005815
A_23_P149345	2,71	down	1,56583	up	PTPN22	Protein tyrosine phosphatase	NM_015967
A_23_P50399	1,22	down	1,5416	up	DCAF15	DDB1 and CUL4 associated factor 15	NM_138353
A_24_P256830	1,18	down	1,6193	up	EXOC5	Exocyst complex component 5	NM_006544
A_23_P360769	1,06	down	1,5956	up	MAN2A1	Mannosidase	NM_002372
A_24_P258473	1,13	down	1,9839	up	SMOC1	SPARC related modular calcium binding 1	NM_001034852
A_23_P40240	1,32	down	2,7139	up	CTSZ	Cathepsin Z	NM_001336
A_23_P214011	2,14	down	1,7433	up	CDH6	Cadherin 6	NM_004932
A_24_P62659	2,08	down	1,6252	up	TSPAN2	Tetraspanin 2	NM_005725
A_24_P147910	1,03	down	1,6621	up	SEPT9	Septin 9	NM_006640
A_24_P363408	5,16	down	1,7213	up	HEY2	Hairy/enhancer-of-split related with YRPW motif 2	NM_012259
A_23_P32577	2,35	down	1,5450	up	DACH1	Dachshund homolog 1 (Drosophila)	NM_080759
A_23_P356139	1,73	down	1,5485	up	FAM178A	Family with sequence similarity 178	NM_018121
A_24_P243749	2,06	down	1,5197	up	PDK4	Pyruvate dehydrogenase kinase	NM_002612
A_24_P176409	2,16	down	1,7183	up	ZFP90	Zinc finger protein 90 homolog (mouse)	NM_133458
A_24_P104538	2,01	down	1,5197	up		Nucleosome-remodeling factor subunit BPTF	XM_001726269
A_32_P56392	1,48	down	1,6076	up	RBMX	RNA binding motif protein	NM_002139
A_24_P187218	1,68	down	1,6168	up	PCDH9	Protocadherin 9	NM_020403
A_23_P156907	2,79	down	1,5262	up	SOBP	Sine oculis binding protein homolog (Drosophila)	NM_018013
A_32_P233769	1,07	down	1,5719	up	U.F.		
A_24_P590806	1,14	down	1,5892	up	U.F.		
A_32_P62835	1,34	down	1,8267	up	U.F.		
A_23_P146981	1,14	down	1,7179	up	U.F.		
A_32_P148627	3,82	down	1,6307	up	U.F.		
A_23_P335190	1,62	down	1,5310	up	U.F.		
A_32_P137408	1,08	down	1,8703	up	U.F.		
A_32_P45229	3,25	down	1,8844	up	U.F.		
A_32_P55799	1,03	down	1,8320	up	U.F.		
A_32_P225328	1,78	down	1,6220	up	U.F.		
A_32_P214969	1,17	down	2,1562	up	U.F.		
A_32_P190222	2,05	down	1,9090	up	U.F.		
A_24_P200427	1,28	up	1,5251	down	PAICS	Phosphoribosylaminoimidazole carboxylase	NM_001079525
A_32_P51524	1,63	up	2,4827	down	LOC595101	mRNA; cDNA DKFZp686H21113	CR627362
A_23_P77721	1,00	up	1,6413	down	LOC100131601	cDNA FLJ45059 fis	AK127004
A_24_P927444	1,66	up	1,6194	down	CYTSB	Sperm antigen with calponin homology and coiled-coil domains 1	BC033618
A_32_P72110	2,10	up	1,8706	down	PVR	Poliovirus receptor	NM_006505
A_24_P319369	1,74	up	1,6563	down	F11R	F11 receptor	NM_016946
A_23_P363344	1,14	up	1,5518	down	TPM1	Tropomyosin 1 (alpha)	NM_000366
A_23_P500844	5,60	up	1,9049	down	PDE5α	Phosphodiesterase 5α	NM_001083
A_24_P145035	1,25	up	1,6981	down		Cobalamin synthetase W domain-containing protein 2	AK097639
A_23_P338479	2,73	up	1,5364	down	CD274	CD274 molecule	NM_014143
A_32_P115701	1,22	up	1,6080	down	NARG2	NMDA receptor regulated 2	NM_024611
A_32_P7974	1,73	up	1,6001	down	TDRD10	Tudor domain containing 10	NM_182499
A_23_P52266	1,18	up	1,5171	down	IFIT1	Interferon-induced protein with tetratricopeptide repeats 1	NM_001548
A_24_P416997	3,12	up	1,6076	down	APOL3	Apolipoprotein L	NM_145641
A_24_P945262	1,55	up	2,7229	down	CARD11	cDNA FLJ39820 fis	AK097139
A_32_P215143	4,40	up	4,7029	down	LOC100288583	Hypothetical protein LOC100288583	XM_002343463
A_24_P148261	2,07	up	2,0703	down	TGF-β2	Transforming growth factor-β2	NM_001135599
A_23_P35995	1,93	up	1,9822	down	ASAM	Adipocyte-specific adhesion molecule (ASAM)	NM_024769
A_32_P25823	1,52	up	1,5729	down	VPS41	Vacuolar protein sorting-associated protein 41 homolog	BX648347
A_23_P343927	2,43	up	1,5286	down	HIST2H2AB	Histone cluster 2	NM_175065
A_32_P8351	1,74	up	2,2334	down	LOC595101	mRNA; cDNA DKFZp686H21113	CR627362
A_24_P829261	1,00	up	3,1194	down	MALAT1	Metastasis associated lung adenocarcinoma transcript 1	NR_002819
A_32_P62769	1,59	up	1,7918	down	LOC100288933	PREDICTED: hypothetical LOC100288933	XR_078366
A_23_P211080	2,99	up	1,6303	down	IFNAR2	Interferon alpha receptor	NM_207585
A_23_P59691	1,53	up	1,6471	down	PAX4	Paired-box transcription factor	AF043978
A_24_P11737	1,06	up	1,6010	down	LOC440894	cDNA FLJ31522 fis	AK056084
A_24_P406132	1,04	up	1,6177	down	MAPK13	mitogen-activated protein kinase 13	NM_002754
A_24_P942703	3,54	up	2,8578	down	LOC728153	cDNA FLJ11903 fis	AK021965
A_24_P450372	1,37	up	2,0113	down	U.F.		
A_24_P937582	1,20	up	1,9464	down	U.F.		
A_24_P450493	5,08	up	1,5469	down	U.F.		
A_23_P22978	1,29	up	1,5873	down	U.F.		
A_24_P938516	1,08	up	1,5411	down	U.F.		
A_24_P649829	1,22	up	1,5841	down	U.F.		
A_24_P59485	2,58	up	1,6852	down	U.F.		

F.C. = fold change; U.F. = Genes of unknown function.

Genes regulated antagonistically with IL-1β.

**Table 2:** Genes differentially expressed in DHA plus IL-1β- versus IL-1β-treated cells.

Probe Name	FC (IL-1 vs None)	Regulation	FC (DHA plus IL-1 vs IL-1)	Regulation	Symbol	Description	Genbank Accession
A_23_P139339	1,12	down	1,7013	down	PAAF1	Proteasomal ATPase-associated factor 1	NM_025155
A_23_P58117	1,76	down	1,9171	down	SHROOM3	Shroom family member 3	NM_020859
A_24_P237601	2,28	down	1,5399	down	RPS6KA5	Ribosomal protein S6 kinase	NM_182398
A_23_P87013	1,12	down	1,6039	down	TAGLN	Transgelin	NM_001001522
A_32_P129752	1,57	down	1,7424	down	TMEM30B	Transmembrane protein 30B	NM_001017970
A_23_P57547	1,16	down	1,5709	down	SLC25A17	Solute carrier family 25	NM_006358
A_23_P70007	1,08	down	1,6774	down	HMMR	Hyaluronan-mediated motility receptor (RHAMM)	NM_012484
A_23_P6818	1,14	down	1,6614	down	SEMA3G	Sema domain	NM_020163
A_23_P113393	1,68	down	1,5961	down	APLN	Apelin	NM_017413
A_23_P135990	1,44	down	1,6862	down	SLCO2A1	Solute carrier organic anion transporter family	NM_005630
A_23_P127891	1,66	down	1,6109	down	BDNF	Brain-derived neurotrophic factor	NM_170735
A_32_P181061	2,39	down	2,1453	down	U.F.		
A_23_P434040	2,23	down	2,4356	down	U.F.		
A_32_P53183	1,59	down	1,5028	down	U.F.		
A_24_P297302	2,53	down	1,7212	down	U.F.		
A_24_P84370	1,02	down	1,5995	down	U.F.		
A_23_P215505	1,90	up	2,6764	up	RAPGEF5	Rap guanine nucleotide exchange factor (GEF) 5	NM_012294
A_24_P133933	1,11	up	1,7661	up	ARHGDIA	Rho GDP dissociation inhibitor (GDI) alpha	NM_004309
A_24_P525917	1,57	up	1,5075	up	NFATC2	Nuclear factor of activated T-cells	Q13469
A_24_P234838	1,37	up	1,5085	up	PCDH1	Protocadherin 1	NM_032420
A_24_P366994	1,03	up	1,9132	up	ARHGDIA	Rho GDP dissociation inhibitor (GDI) alpha	NM_004309
A_23_P169738	1,19	up	1,5391	up	SOX7	SRY (sex determining region Y)-box 7	NM_031439
A_23_P218807	2,23	up	1,7046	up	ZC3H7B	Zinc finger CCCH-type containing 7B	NM_017590
A_23_P96165	1,04	up	1,6745	up	C11orf80	Chromosome 11 open reading frame 80	NM_024650
A_23_P202435	1,02	up	1,6325	up	ADD3	Adducin 3 (gamma)	NM_016824
A_32_P165477	1,50	up	1,5528	up	SLC7A11	Solute carrier family 7	NM_014331
A_32_P55241	2,88	up	1,5342	up	SHISA2	Shisa homolog 2 (Xenopus laevis)	NM_001007538
A_23_P112078	1,75	up	1,5282	up	MFHAS1	Malignant fibrous histiocytoma amplified sequence 1	NM_004225
A_32_P20040	1,58	up	1,7230	up	U.F.		
A_24_P911327	1,03	up	1,5692	up	U.F.		
A_32_P62480	1,25	up	1,9490	up	U.F.		
A_32_P222250	1,53	up	1,6226	up	U.F.		
A_32_P201976	1,43	up	1,7541	up	U.F.		
A_32_P48149	1,13	up	1,5691	up	U.F.		
A_24_P366989	1,01	up	1,8311	up	U.F.		
A_32_P229365	1,19	up	1,6773	up	U.F.		
A_24_P85619	1,16	up	1,5773	up	U.F.		
A_32_P30898	1,08	up	1,5220	up	U.F.		
A_32_P174374	1,02	up	2,0840	up	U.F.		
A_32_P112359	1,08	up	1,7697	up	U.F.		
A_32_P151648	1,10	up	1,5138	up	U.F.		

F.C. = fold change; U.F. = Genes of unknown function.

Genes regulated agonistically with IL-1.

**Fig 3:**
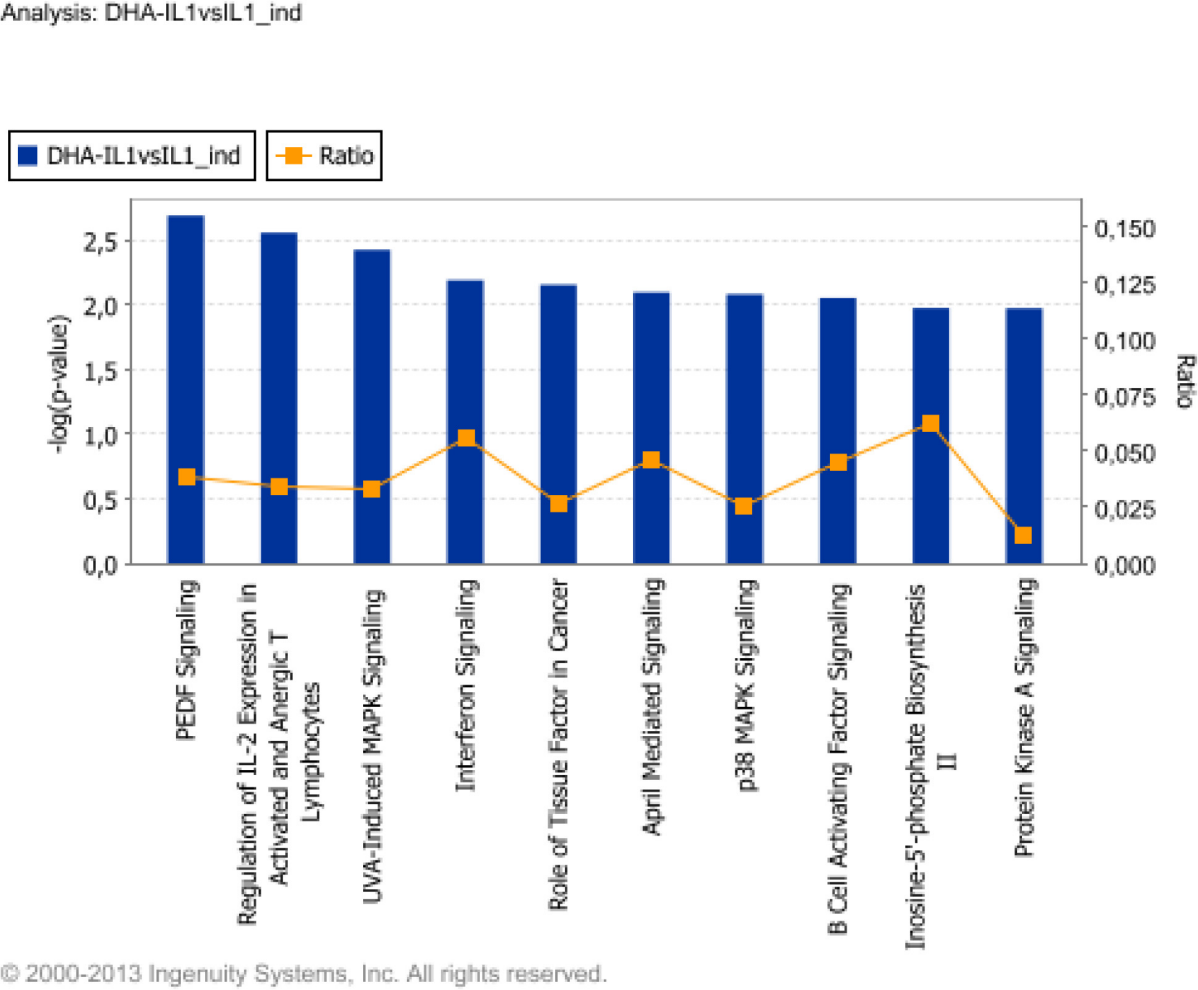
Top ten signaling and metabolic pathways regulated by interleukin (IL)-1β upon cell treatment with DHA. For the functional categorization of genes, Fischer’s exact test was used to calculate a P value (shown as bars), indicating the probability that each biological function assigned to the network is due to chance alone. The ratio (shown as squares) represents the number of differentially expressed genes in a given pathway divided by total number of genes making up that canonical pathway.

When classified by their function, these genes resulted associated with biological process including cancer, cell growth and proliferation, nervous system development and function, tissue development, and cardiovascular system development and function. Among such biological processes, most genes affected by DHA were related to breast and digestive system cancer (32 molecules), cell proliferation (28 molecules), and morphology of the cardiovascular system (10 molecules) (Table F in S2 File). IPA identified assorted canonical pathways associated with these genes. Ranked according to their significance (P value), they included: pigment epithelium-derived factor (PEDF) signaling, regulation of IL-2 expression in activated and anergic T lymphocytes, ultra-violet A (UVA)-induced MAPK signaling, interferon signaling, the role of tissue factor in cancer¸ proliferation-inducing ligand (APRIL)-mediated signaling, and p38 MAPK signaling (Fig 3). Genes included in each group of the top ten signaling pathway are listed in Table G in S2 File. When IPA was enquired for network analysis, it yielded 6 significant regulatory networks (score>2). The first ranked network (score = 38, focus molecules = 18) (S3 Fig) was associated with cellular function and maintenance, humoral immune response and protein synthesis. Top functions of the other 5 highly significant networks were associated with drug metabolism, cell death and survival, organ morphology and cardiovascular system development and function.

### CD47 and CARD11 functional analysis

To best define the functional role of some genes affected by DHA in endothelial cells, we elected to silence the expression of two of them, CARD11 and CD47, and then to evaluate the resulting effects in terms of expression of genes potentially correlated. We selected CARD11 and CD47 because both were hypothesized to be involved in the signaling pathway leading to the activation of the pro-inflammatory master transcriptional factor nuclear factor(NF)-κB [15]. Transfections with siRNAs neither affected cell morphology, nor produced overt signs of endothelial cell damage (S4A and S4B Fig). Analysis of CD47 and CARD11 gene expression after silencing showed a significant and specific decrease in the levels of both mRNAs, as analyzed by qRT-PCR, by >70% and >80% under basal and stimulated condition for CD47 and CARD11, respectively (S4C Fig). Under the same conditions the levels of several pro-inflammatory and vascular remodeling genes were assessed. CARD11 silencing blunted the IL-1β-mediated expression of most pro-inflammatory genes tested, including vascular cell adhesion molecule (VCAM)-1, intercellular adhesion molecule (ICAM)-1, and E-selectin (E-Sel) (S5 Fig), likely through an attenuation of the NF-κB signaling pathways [16]. Interestingly, CARD11 knockdown also downregulated the expression of genes encoding for vascular endothelium growth factor (VEGF) and matrix metalloproteinase(MMP)-2, and increased the expression of the potent anti-inflammatory cytokine IL-10 [17] (S5 Fig), thus disclosing new unexpected functions for this gene. Finally, we observed that CD47 knockdown reduced the expression of all the pro-inflammatory, pro-angiogenic genes tested, confirming in our experimental conditions the pro-inflammatory role previously ascribed to this molecule [18] (S6 Fig).

### Microarray analysis verification and validation

**Fig 4:**
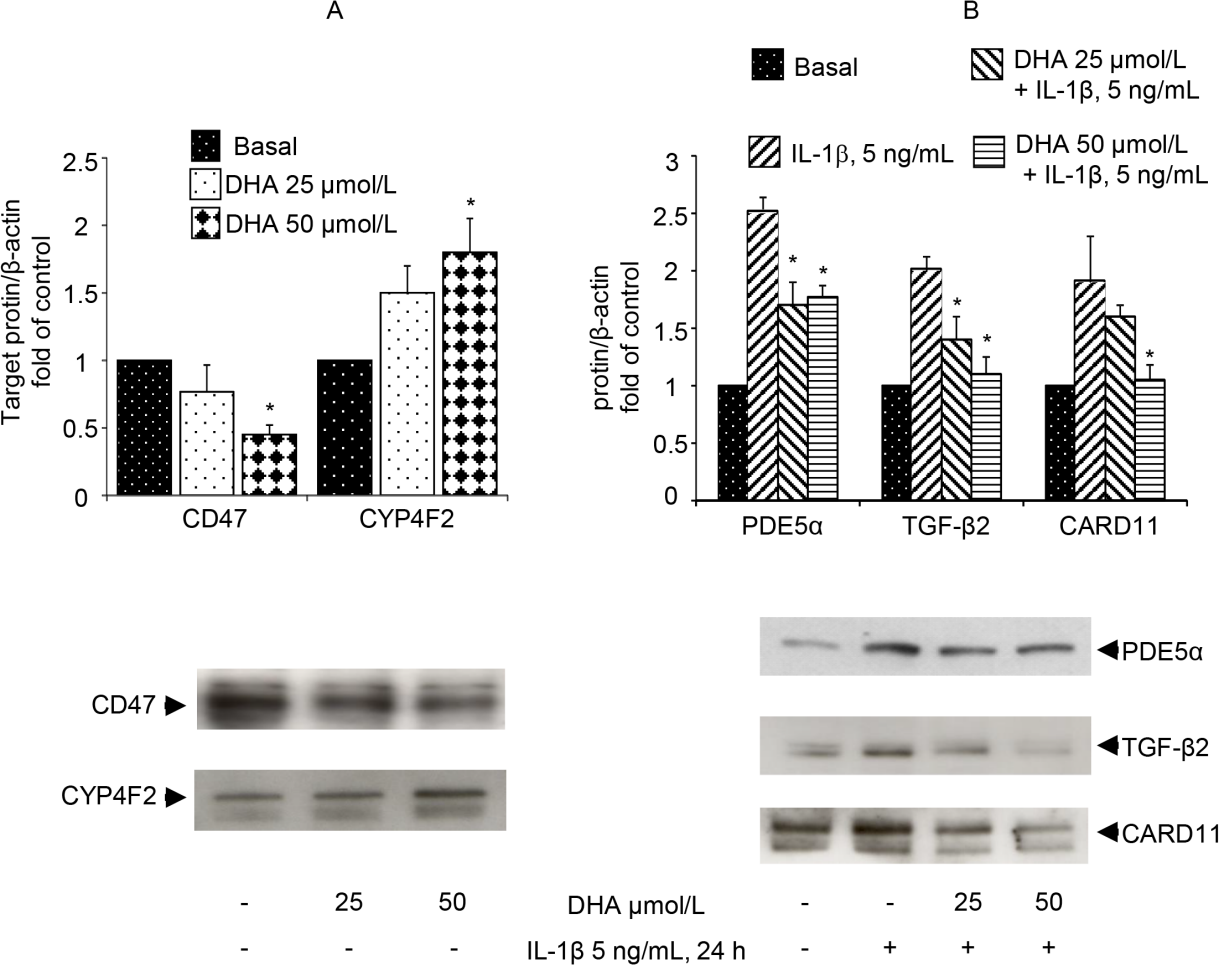
Validation of gene expression changes in DHA-treated HUVECs by Western blotting. (A) HUVECs were treated with DHA for 48 h, after which time total protein were extracted and subjected to Western analysis using an anti-CD47 and an anti-CYP4F2 antibody. As control for equal loading, blots were stripped and re-probed with an anti-β-actin antibody. CD47 and CYP4F2 representative immunoblots are shown in the lower panels, while densitometric analysis for the CD47 and CYPFF2, normalized to β-actin and expressed as fold induction over basal control, are shown in the upper panel. Bars represent mean±SD (n = 3). *P<0.05 vs basal control. (B) HUVECs were treated with/without DHA for 48 h, and then stimulated with IL-1β. After 24 h, total protein were extracted and subjected to Western analysis using antibodies against PDE5α, TGF-β2 and CARD11. As control for equal loading, blots were stripped and re-probed with an anti-β-actin antibody. Representative immunoblots are shown in the lower panels, while densitometric analysis for each protein, normalized to β-actin and expressed as fold over basal control, are shown in the upper panel. Bars represent mean±SD (n = 3). *P<0.05 vs IL-1β.

We used qRT-PCR and Western analysis to validate the microarray results for a subset of genes selected from the DHA- plus IL-1β and the DHA+ plus IL-1β experimental conditions. Target genes were chosen based upon their potential vascular regulatory functions. Among those deregulated by DHA under resting conditions, we focused our attention on the modulation of CD47 and CYP4F2 expressions. For these two genes, microarray analysis showed a down-regulation by 1.5 FC and an up-regulation by 2.52 FC, respectively. In agreement with microarray data, qRT-PCR confirmed the reduction of CD47 and the increase of CYP4F2 mRNA expressions (-40 ± 9% and +30 ± 5%, respectively, for 50 μmol/L DHA; p<0.01). Western analysis confirmed such findings in terms of protein expression (Fig 4A). Exposure of HUVECs to IL-1β induced the expression of several well-known pro-inflammatory genes, among which those encoding for VCAM-1 (27.7 FC), for the chemokine (C-C motif) ligand(CCL)2 (also referred to as monocyte chemotactic protein (MCP)-1) (3.78 FC) and the prostaglandin-endoperoxide synthase (PTGS)-2 (also known as cyclooxygenase(COX)-2) (1.8 FC), in addition to a vast series of new unexplored genes of cardiovascular interest, such as those encoding for transforming growth factor (TGF)-β2 (2.2 FC) and phosphodiesterase (PDE)5α (5.6 FC). Analysis by qRT-PCR confirmed microarray results showing the induction of VCAM-1, MCP-1, COX-2, TGF-β2 and PDE5α upon IL-1β stimulation by 400-, 6.5-, 6-, 5- and 30-fold, respectively. We also confirmed microarray results obtained in cells exposed to DHA before IL-1β stimulation in comparison with IL-1β stimulation alone. Under these conditions, microarray analysis showed, for the DHA plus IL-1β condition, a down-regulation of TGF-β2, PDE5α and CARD11 by 2.0 and 1.9 and 2.72 FC, respectively. Such expression changes were confirmed at both qRT-PCR (-35%, -37% and -25%; respectively, S7 Fig) and Western analysis (Fig 4B).

We noticed that several other NF-κB-target genes, including VCAM-1, MCP-1 and COX-2, robustly induced by IL-1β and previously described as affected by DHA [14, 19], did not result down-regulated by DHA in the present microarray analysis. It must however be recognized that conclusions drawn from the comparison of gene expressions under two treatments with this technique are strictly dependent upon the selection of significance criteria. In microarray analyses, these are usually set at values aimed at achieving a robust degree of specificity at the price of sensitivity. Therefore it is well possible that genes the expression of which does not achieve the pre-defined threshold are here classified as unchanged, when in reality expression level changed. In our analysis this was true, for example, for VCAM-1, MCP-1, COX-2, ICAM-1 and E-Sel, the induced expression of which, even resulting as not affected by DHA at the microarray analysis, still resulted down-regulated at qRT-PCR analysis (S7 Fig), a technique widely recognized as of greater sensitivity than microarray [20, 21], and here run in parallel with a few a priori selected genes.

## Discussion

This study reports on the changes in global gene expression in human endothelial cells exposed to DHA under resting and pro-inflammatory conditions. Besides confirming gene regulations previously assessed with a classical candidate-gene approach, this analysis discloses previously unsuspected genes and gene pathways affected by the exposure induced of endothelial cells to DHA, thus expanding our knowledge on biological effects of n-3 PUFAs.

Although there is still no unanimous consensus on the vasculoprotective role of n-3 PUFAs [22], a significant number of experimental studies and clinical intervention trials have shown a protective effect for these substances derived from the intake of dietary fish or fish oil [1]. However, the probably multiple molecular mechanisms by which EPA and DHA affect cardiovascular health still remain incompletely understood [1]. To obtain a comprehensive overview of the scope of biological processes modulated by n-3 PUFAs whole-genome transcriptomic analyses have been previously performed on peripheral blood mononuclear cells (PBMCs) of human subjects taking fish oils supplements, with results suggesting a modulation by n-3 PUFAs of inflammatory-, oxidative-, endoplasmic reticulum (ER) stress- and apoptosis-regulatory pathways [21, 23–27]. However, studies examining the effects of n-PUFAs on whole-genome expression of endothelial cells, which are prominent targets for n-3 PUFA effects [1], have been lacking. Our analysis on these cells had two main objectives: firstly, to explore the effects of DHA supplementation on changes in endothelial gene expression under resting conditions; and secondly, to evaluate the effects of DHA on changes in endothelial gene expression under pro-inflammatory conditions. This latter goal was accomplished comparing the gene expression profile of IL-1β-stimulated cells with those of cells treated with DHA before IL-1β stimulation.

Fixing rather stringent analytical conditions of FC and significance (cut-offs >1.5, P values <0.05), under unstimulated conditions we identified 188 genes as differentially expressed by DHA. Taking advantage of ranked gene expression pathways, the results revealed that pathways related to stemness, cell-to-cell signaling and inflammation were among those mostly differentially expressed. In particular, the canonical pathway analysis revealed the “role of Nanog in mammalian embryonic stem cell pluripotency” as the most significant signaling pathway modulated by DHA. This is an example of an unexpected and interesting result. The possibility that DHA regulates the expression of stemness transducers may provide an opportunity to use DHA and n-3 PUFAs in general to enhance the generation of progenitor cells or stem-like cells and enhance reparative functions of endothelial progenitor cells (EPCs). Despite the paucity of experimental data on the effect of n-3 PUFAs on EPC function, some recent reports support this hypothesis [28, 29].

A classification of DHA-regulated genes into functional related groups revealed that DHA per se maximally impacts gene expression connected with cancer initiation and progression (best scored function annotation). However, although less scored, we also focused our attention on activities connected with cardiovascular system development and functions. The most cited genes gathered under this category are involved in the morphology of cardiovascular system and the regulation of blood pressure, and include TGF-β2, ANGPT-1, CD47 and CYP4F2, the first three of which are down- regulated, while the last is up-regulated by DHA. The TGF-β family includes a large number of molecules structurally and functionally related, acting as multifunctional regulators of a wide range of biological processes, including morphogenesis, embryonic development, adult stem cell differentiation, immune regulation, wound healing, inflammation, atherogenesis and cancer [30]. Despite their high sequence homology, striking differences exist between the 5’flanking regions of each such genes. TGF-β1 has not a TATAA box nor nuclear factor (NF)-κB binding sites, otherwise present in the TGF-β2 promoter sequence [31, 32]. Furthermore, while TGF-β1 and -β3 bind directly to the TGF-β receptor type II (TβRII), the binding of TGF-β2 requires the presence of a co-receptor, which may explain differences in activities of TGF-β2 and -β1 [33]. TGF-β1 is the isoform most extensively studied in relation to atherogenesis, with somewhat contrasting results [34, 35]. More pertinently to our results, it was demonstrated that TGF-β2 may specifically mediate neointimal thickening in LDLr^-/-^ mice after carotid artery ligation [36], and induces the activation of pro-inflammatory transcription factors, such as NF-κB, in various cellular models [37]. Furthermore, a synergistic cross-talk between TGF-β2 and IL-1β in the activation of NF-κB has been also documented [38]. In line with this pro-inflammatory cross-talk, we observed that IL-1β could induce TGF-β2, but not TGF-β1 expression; and that DHA treatment reduced both the basal and the up-regulated expression of TGF-β2, thus revealing another mechanistic explanation for the anti-inflammatory activities attributed to DHA. The mechanism by which DHA may reduce basal and IL-1β mediated expression of TGF-β2 goes beyond the aim of the present report and was not investigated further. However, since DHA is known to negatively interfere with the activation of the transcription factor NF-κB in the vascular endothelium [19], the selective presence of a NF-κB binding site in the TGF-β2 promoter sequence [32] may help explaining both the preferential induction of TGF-β2 by IL-1β and the down-regulation of TGF-β2 by DHA. Previous reports investigating the effect of DHA on TGF-β2 expression are scarce. However, in line with our results, the expression of TGF-β2 was reduced in platelets of rats fed on a diet high in fish oils [39].

Interestingly, TGF-βs are extracellularly activated by the catalytic activity of trombospondin (TSP)-1, the receptor for which, CD47, also resulted down-regulated by DHA. TSP-1/CD47 interaction has been reported to exert pro-inflammatory activities in the vascular endothelium, inducing the expression of pro-atherogenic adhesion molecules and the subsequent binding of monocytes [18] and T-cell [40], and to negatively interfere with the nitric-oxide (NO)-driven vascular smooth muscle cell (VSMC) relaxation [41], and, as a consequence, affecting local and systemic blood flow [42]. We here confirm the involvement of CD47 in the orchestration of endothelial pro-inflammatory response to IL-1β, thus expanding the findings obtained by Narizhneva at al. upon endothelial exposure to TNF-α [18]. We observed that DHA reduces the basal expression of CD47, thus potentially contributing to the small but significant decrease in blood pressure generally ascribed to fish oil intake [43]. While a down-regulating effect of DHA on TSP-1 has been recently reported in adipocyte-macrophages co-cultures [44], our investigation is the first reporting a down-regulating activity by DHA on the expression of the TSP-1 receptor CD47. Finally, among genes regulated by DHA under basal conditions, we observed the up-regulation of CYP4F2, a member of the cytochrome P 450 gene 4 family. In general, human CYP comprises a superfamily of heme-thiolate proteins that play critical roles in the metabolism of endogenous as well as xenobiotic-derived molecules [45]. Families from 1, 2 and 3 are known to be involved in the epoxidation of drugs and of other xenobiotics in the liver [46], whereas the CYP4 –CYP4Fs in particular–are known to n-hydroxylate a variety of long-chain unsaturated and branched-chain FA, vitamins with long alkyl side chains, leukotrienes (LT), prostaglandins (PG) and hydroxyeicosatetraenoic acids (HETE) [46]. CYP4F was first discovered to metabolize and inactivate the pro-inflammatory leukotriene (LT) B_4_ [47]. Recent reports have shown the expression of CYP4F2 and CYP4F3 genes in the colon of patients with inflammatory celiac disease [48]. In particular, the CYP4F3 gene resulted overexpressed during cryptic hyperplasia, while in remission stages a strong increase in CYP4F2 gene expression was observed [49]. These data suggest that CYP4F3 is involved in promoting colon inflammation, while the increased expression of CYP4F2 would mediate disease resolution. CYP4F2 has been also recognized to metabolize arachidonic acid (AA) to 20-HETE, a molecule endowed with anti- and pro-hypertensive properties [50]. Since DHA may be used by CYP4F2 as substrate alternative to AA [51], DHA may exert anti-inflammatory and anti-hypertensive effects by both decreasing the AA conversion to 20-HETE and decreasing LTB_4_ levels. The ability of DHA to interfere with the CYP4F-metabolic pathway through genomic ways is exciting, and deserves further investigations.

IL-1 β has long been considered to play a central role in orchestrating the various cellular changes that contribute to inflammation in atherogenesis [52]. In humans IL-1β expression is higher in atherosclerotic lesions than in normal arteries [53]. Studies in mouse models of atherosclerosis have shown a reduced atherosclerotic burden in IL-1β-deficient animals [54]. For these reasons we chose to challenge the endothelium with IL-1β as an inflammatory trigger to evaluate the interference by DHA with endothelial activation. In agreement with previous DNA microarray studies [55–57], the gene expression profile analysis here revealed complex inflammatory responses comprised of up- and down- regulation of genes connected with several cellular processes, including cell development, cell death and survival, and cardiovascular system development and function (this last as the 5^th^ best scored change). Among the up-regulated genes, our microarray data confirm well-validated changes including COX-2, IL-8, MCP-1, IL-6, E-selectin, VCAM-1, ICAM-1, and fractalkine, all reported as IL-1β up-regulated genes in previous microarray studies [56–59].

As a second aim of our study, among the confirmed IL-1β-up-regulated genes, we then focused our attention on those of relevance for atherosclerosis and maximally counter-impacted by DHA treatment. Some of these genes were grouped in known canonical pathways, such as TGF-β2 and PDE5 associated with protein kinase A signaling; and CARD11, associated with the regulation of IL-2 expression in activated and anergic T Lymphocytes; while others, such as F11R, were not connected with any canonical pathways.

F11R, also known as junctional adhesion molecule (JAM)-A, is a small multifunctional immunoglobulin expressed by platelets, leukocytes, endothelial and epithelial cells, acting as a ligand for the integrin lymphocyte function-associated antigen (LFA)-1 during leukocyte transmigration [60], a platelet receptor [61], and a receptor for rheoviruses [62]. A prominent role for F11R in several inflammatory pathologic processes, including skin inflammation, meningitis, peritonitis, liver and myocardial ischemia, and coronary artery disease, has been recently recognized [63]. In particular, a significant increase in the level of soluble F11R has been found in the serum of patients with coronary artery disease [64]; and significantly higher levels of the F11R mRNA and protein are expressed within unstable atherosclerotic plaques in association with the endothelium and platelets [65], suggesting the involvement of F11R in atherothrombosis. *In vitro* and *in vivo* studies have shown that, under physiological conditions, a non-activated, healthy endothelium expresses low levels of F11R mRNA, and in this case the F11R/JAM-A protein resides primarily within the endothelial tight junctions [66]. When endothelial cells are exposed to pro-inflammatory cytokines, such as IL-1β and tumor necrosis factor (TNF)α, mRNA and protein levels for F11R rise significantly, followed by the insertion of newly-synthesized F11R molecules into the endothelial plasma membrane facing the vessel lumen [67], thus providing the endothelium with new pro-thrombotic and pro-adhesive properties. In agreement with previous data [68], we observed an increase in F11R mRNA expression upon IL-β stimulation. We also observed that DHA pretreatment antagonizes IL-1β-mediated increase in mRNA levels of F11R, an anti-inflammatory effect never reported before, highlighting potentially new mechanistic explanations for the anti-atherogenic and anti-thrombotic effects of DHA. The presence of NF-κB binding sites in the promoter of the F11R gene [69] may explain the down-regulating effect exerted by DHA, confirming for DHA the repeatedly described interference with the expression of NF-κB target genes [19]. NF-κB activation indeed represents one of the foremost mechanisms responsible for IL-1β-induced endothelial activation. Similarly to many activators of NF-κB, this occurs through the so-called canonical pathway, whereby activation depends on stimulation of the IκB kinase (IKK) complex. IKK in turn orchestrates the phosphorylation and subsequent degradation of IκB, a protein that sequesters NF-κB (particularly in the form of RelA/p65) in the cytoplasm. Upon IκB degradation, NF-κB is then free to translocate to the nucleus and stimulate transcription of various pro-inflammatory genes [15, 70]. In our previous work on the mechanisms by which DHA interferes in the signaling leading to NF-κB activation, we have shown that DHA inhibits at least two molecular switches involved in NF-κB activation, namely NAD(P)H oxidase and PKCε [19]. The present data suggest an additional level of interference in the signaling pathway leading to NF-κB activation. We here observed that IL-β induced, and DHA counter-reduced, the expression of the Caspase recruitment domain (CARD)11 mRNA, which encodes for a scaffold protein involved, upon trimerization with B-cell lymphoma protein 10 (Bcl10) and Mucosa-associated lymphoid tissue lymphoma translocation protein 1 (MALT1), in the activation of IKK [71]. In spite of generally reported tissue-specific expression of CARD11 in hematopoietic cells [16], a recent gene expression analysis has recognized the expression of CARD11 also in endothelial cells and its induction in the endothelium within a hemangioma [72]. To the best of our knowledge, this is the first report describing an induction of CARD11 by IL-1β in endothelial cells and its direct involvement in the induction of pro-inflammatory genes. The down-regulation of IL-1β-induced expression of CARD11 by DHA is therefore an additional mechanistic explanation underpinning the anti-inflammatory effect exerted by n-3 PUFAs. Endothelial dysfunction, reflected by reduced NO availability, is recognized as a causal factor in promoting atherosclerosis [73]. Synthesized from L-arginine through the catalysis of a family of NO synthase isoforms, NO activates the soluble guanylate cyclase (sGC) and the subsequent generation of cGMP, which in turn, by activating protein kinase (PK)G, serves as a final modulator of vascular relaxation and platelet aggregation [74]. N-3 PUFAs have been shown to increase endothelium-dependent vasodilation in coronaries arteries of normal pigs [75] and in isolated rat aortic ring preparations by enhancing the release of NO [76]. Our current data now suggest an additional interference by DHA in the NO-sGC-cGMP axis. Among the most cited genes up-regulated by IL-1β and down-regulated by DHA, we found the one encoding for PDE5. This enzyme selectively catalyzes the hydrolysis of cGMP into GMP, thus curtailing NO signaling [77]. The importance of cGMP in NO signaling has encouraged, in the past years, the development of PDE5 inhibitors, the first one being sildenafil [78]. Nowadays, PDE5 inhibitors have approved indications for their use in erectile dysfunction and pulmonary hypertension, and have a potential in the treatment of pathological states featuring endothelial dysfunction, including diabetes and the metabolic syndrome, heart failure and Raynaud’s phenomenon [79]. DHA appears in many aspects to reproduce the therapeutic potential of PDE5 inhibitors, down-regulating PDE5 mRNA levels induced by IL-1β. To the best of our knowledge, no previous report had described activation of the PDE5 gene expression by IL-1β, or the related down-regulation by n-3 PUFAs. The presence of Sp1, AP-1 and AP-2 binding sites in the promoter sequence of PDE5 [80] may account for both PDE5 mRNA induction by IL-1β and the modulatory activity exerted by DHA [81, 82].

In conclusion, the data presented here yield novel information on cardiovascular health effects linked to n-3 PUFAs and fish oil consumption. They demonstrate that exposure of human endothelial cells to DHA causes deep changes in gene expression that may prevent endothelial dysfunction and protect the endothelium from the occurrence of atherosclerosis under basal and, more relevant, under pro-inflammatory conditions. Several pathways here shown to be regulated by DHA are new, and suggest promising reinterpretations of the therapeutic potential of n-3 PUFAs and of their role in modulating activity and function of EPC and stem cells. Being ours, to the best of our knowledge, the first report in the specific endothelial research area, we were unable to produce comparative evaluations with similar literature reports. However our data point out to a global interference by DHA with the expression of NF-κB target genes, as well as with modulators of such activation, confirming and expanding the anti-inflammatory and anti-atherogenic activities highlighted in vivo [21, 23, 25, 26] and providing new molecular explanations to evaluate the role of fish oil-derived n-3 PUFAs in the context of diseases featuring endothelial dysfunction.

## Supporting Information

S1 File Supporting materials and methods. (DOC)

S2 File Supporting Tables. (XLS)

S1 Fig The most significant network regulated by DHA in resting unactivated conditions. Networks of genes were algorithmically generated with the IPA software based on their connectivity and assigned a score. The intensity of the node color indicates the degree of up- (red) or down-(green) regulation. A continuous line means a direct relationship between the two genes, whereas a discontinuous line indicates an indirect association. The network depicted includes 22 focus molecules.
(TIF)

S2 Fig Top ten signaling and metabolic pathways regulated by IL-1β as compared to unstimulated conditions. For the functional categorization of genes, Fischer’s exact test was used to calculate a P value (shown as bars) indicating the probability that each biological function assigned to the network is due to chance alone. The ratio (shown as squares) represents the number of differentially expressed genes in a given pathway divided by total number of genes that make up that canonical pathway
(TIF)

S3 Fig The most significant network regulated by DHA in IL-1β stimulated conditions. Networks of genes were algorithmically generated with the IPA software based on their connectivity and assigned a score. The intensity of the node color indicates the degree of up- (red) or down-(green) regulation. A continuous line means a direct relationship between the two genes, whereas a discontinuous line indicates an indirect association. The network depicted includes 18 focus molecules.
(TIF)

S4 Fig siRNA-mediated CD47 and CARD11 knockdown. HUVECs were transfected with non-silencing (siControl), CD47 or CARD11 siRNA for 72 before IL-1β stimulation. (A) Phase contrast images of the endothelial monolayers after silencing of CD47 and CAR11. Bars, 20 μm. (B) Effect of Cd47 and CARD11 silencing on endothelial vitality. After gene silencing and IL-1β stimulation MTT assay was performed. In no culture condition tested were highlighted evidence of toxicity by gene silencing. Absorbance data are expressed as milliunits (mU), mean ± standard deviation (S.D.) (n = 16). (C) HUVEC were transfected with CD47 siRNA, CARD11 siRNA or non-silencing siRNA (siControl) for 72 h before 3 h stimulation with 5 ng/mL IL-1β. CD47 and CARD11 mRNA expression levels were analyzed by qRT-PCR. Data are expressed as fold induction over unstimulated siControl and derive from three independent experiments performed in duplicate. **<P<0.01 between groups joined by the horizontal lines.
(TIF)

S5 Fig CARD11 functional analysis. HUVEC were transfected with CARD11 siRNA or nonsilencing siRNA (siControl) for 72 hours. After 3 h stimulation with 5ng/mL IL-1β total RNA was isolated and gene expression profile of the indicated genes assessed by qRT-PCR. Data are presented as fold induction over siControl + IL-1β and derive from two independent experiments performed in duplicate. *P<0.01 vs siControl + IL-1β.
(TIF)

S6 Fig CD47 functional analysis. HUVEC were transfected with CD47 siRNA or nonsilencing siRNA (siControl) for 72 hours. After 3 h stimulation with 5 ng/mL IL-1β total RNA was isolated and gene expression profiles of the indicated genes assessed by qRT-PCR. Data are presented as fold induction over siControl + IL-1β and derive from two independent experiments performed in duplicate. *P<0.05 vs siControl + IL-1β; **P<0.0 vs siControl + IL-1β.
(TIF)

S7 Fig Validation of gene expression changes in HUVECs treated with DHA before IL-1β stimulation by qRT-PCR. HUVECs were treated with/without DHA for 48 h and then stimulated with IL-1β. After 3 h mRNA level of various genes was assayed by qRT-PCR. Data are expressed as fold induction over unstimulated control and derive from three independent experiments performed in duplicate. *P<0.05 vs IL-1β alone; **P<0.01 vs IL-1β alone. PDE5α, phosphodiesterase 5α; TGF-β2, transforming growth factor-β2; CARD11, caspase recruitment domain-11; COX-2, cyclooxygenase-2; VCAM-1, vascular cell adhesion molecule-1; ICAM-1, intercellular adhesion molecule-1; E-Sel, E-selectin; MCP-1, monocyte chemoattractant protein-1.
(TIF)
