# Correction: Transcriptome-Based Identification of New Anti-Anti-Inflammatory and Vasodilating Properties of the n-3 Fatty Acid Docosahexaenoic Acid in Vascular Endothelial Cell Under Proinflammatory Conditions

**DOI:** 10.1371/journal.pone.0154069

**Published:** 2016-04-14

**Authors:** Marika Massaro, Rosanna Martinelli, Valentina Gatta, Egeria Scoditti, Mariangela Pellegrino, Maria Annunziata Carluccio, Nadia Calabriso, Tonia Buonomo, Liborio Stuppia, Carlo Storelli, Raffaele De Caterina

In the title of this article, “anti-” incorrectly appears twice instead of once. The correct title is: Transcriptome-based identification of new anti-inflammatory and vasodilating properties of the n-3 fatty acid docosahexaenoic acid in vascular endothelial cell under proinflammatory conditions. The correct citation is: Massaro M, Martinelli R, Gatta V, Scoditti E, Pellegrino M, Carluccio MA, et al. (2015) Transcriptome-based identification of new anti-inflammatory and vasodilating properties of the n-3 fatty acid docosahexaenoic acid in vascular endothelial cell under proinflammatory conditions. PLoS ONE 10(6): e0129652. doi:10.1371/journal.pone.0129652
