# Supplementary material for: Transcriptome-based identification of new anti-anti-inflammatory and vasodilating properties of the n-3 fatty acid docosahexaenoic acid in vascular endothelial cell under proinflammatory conditions
Source: PLoS One. 2015 Jun 26;10(6):e0129652. doi: 10.1371/journal.pone.0129652 (PMC4482638; doi:10.1371/journal.pone.0129652)
Supplement: S1 File — (DOC) [file pone.0129652.s001.doc]

**Transcriptome-based identification of new anti-anti-inflammatory and vasodilating properties of the n-3 fatty acid docosahexaenoic acid in vascular endothelial cell under proinflammatory conditions**

Marika Massaro^1¥^, Rosanna Martinelli^2,3 ¥^, Valentina Gatta^4^, Egeria Scoditti^1^,

Mariangela Pellegrino^1,5^, Maria Annunziata Carluccio^1^, Nadia Calabriso^1^, Tonia Buonomo^2^,

Liborio Stuppia^4^, Carlo Storelli^5^, and Raffaele De Caterina^4,6^

**^1^**C.N.R. Institute of Clinical Physiology, Lecce, Italy

^2^CEINGE Biotecnologie Avanzate, Naples, Italy

^3^Department of Medicine and Surgery of Salerno University, Italy,

^4^“Gabriele d’Annunzio” University and Center of Excellence on Aging, Chieti, Italy

^5^Department of Biological and Environmental Science and Technology (Disteba), University of Salento, Lecce, Italy and

^6^Fondazione Toscana “Gabriele Monasterio”.

Correspondence:

Raffaele De Caterina, M.D., Ph.D. Institute of Cardiology, “G. d’Annunzio” University - Chieti

C/o Ospedale SS. Annunziata – Via dei Vestini - 66013 Chieti, Italy; phone: +39-0871-41512

FAX: +39-0871-402817; E-mail: [rdecater@unich.it](mailto:rdecater@unich.it)

**Materials and Methods**

**Materials**

DHA (22:6 n-3 all cis) was obtained as 99% pure sodium salts from Nu-Chek (Elysian, MN, USA). DHA from two other commercial sources [Calbiochem (La Jolla, CA, USA) and Sigma-Aldrich (St. Louis, MO, USA)] was also used as control. IL-1β was obtained from Sigma. All other reagents were purchased from Sigma.

**Cell isolation and culture**

Human umbilical vein endothelial cells (HUVECs) were isolated from segments of umbilical cords from normal-term deliveries, and cultured in Medium 199 (Life Technologies, Paisley, UK) supplemented with 50 mg/mL retinal growth factor, 10 units/mL heparin, 100 units/mL penicillin, 100 mg/mL streptomycin (Life Technologies), 2 mmol/L L-glutamine (Life Technologies), and 10% fetal calf serum (Life Technologies). Once grown to confluence, cells were re-plated on low-pyrogen 0.1% gelatin-coated culture-grade polystyrene plastic at 20,000 cells/cm^2^. Cells were treated with DHA in complete medium at pre-determined times, with cells at confluence, and up to passage 3. All cultures featured a cobblestone cell morphology and positive staining for von Willebrand factor, as previously reported .

**Cell viability**

Cell viability was determined by MTT assay. Briefly, after the pertinent treatment MTT reagents (3-(4,5-dimethylthiazol-2-yl)-2,5-diphenyl tetrazolium bromide, 0.5 mg/mL) was added to each well for 4 h and the insoluble purple formazan products were dissolved by isopropanol. Absorbance was finally measured spectrophotometrically at 490 nm by a microplate reader.

**Experimental design and RNA extraction**

HUVECs were preincubated with 25 µmol/L DHA for 0-48 h, followed by stimulation with 5 ng/mL IL-1β, for additional 0-3 h, after which time cells were collected and total RNA extracted using the Qiagen RNeasy kit (Qiagen, Milan, Italy) according to manufacturer’s instructions. The eluted total RNA was stored at -80 °C until use. Concentration and purity of RNA was determined by NanoDrop ND-1000 UV-Vis Spectrophotometry (NanoDrop Technologies, Wilmington, DE, USA), while the integrity of RNA was verified using the Agilent 2100 Bioanalyzer (Agilent Technologies Inc., Santa Clara, CA, USA). The same RNAs were used for microarray and real-time polymerase chain reaction (PCR) analysis. Additional real-time (rt) PCR analyses were also performed on different HUVEC samples to further confirm microarray data results. No variation in the total RNA yield was observed under the different experimental conditions tested (data not shown).

**Microarray analysis**

For microarray analysis, RNAs were labeled using the Agilent low RNA Input Fluorescent Linear Amplification kit, and purified with Qiagen’s RNeasy mini spin columns. Differentially labeled RNA samples were co-hybridized on microarray slides. Hybridization was performed using the Gene Expression Hybridization kit (Agilent Technologies) following manufacturer’s instructions. Gene expression profiles were generated using the 4x44K glass slide Whole Human Genome Oligo Microarray G4112A (Agilent Technologies). Each microarray uses 45,220 probes to interrogate 30,886 unique human genes and transcripts. Each array assessed total RNAs from treated endothelial cells (DHA, IL-1 or DHA + IL-1) with RNA obtained from control endothelial cells (untreated endothelial cells). The raw data were processed by GeneSpring 10, as previously described . Microarray data have been uploaded in the Gene Expression Omnibus (GEO) database (<http://www.ncbi.nlm.nih.gov/geo/>) under the following accession number: GSE57825.

**Network identification and canonical pathway analysis**

Lists of genes significantly regulated by DHA and/or IL-1β were analyzed by the Ingenuity Pathways Analysis (IPA) software (Ingenuity Systems, Redwood City, CA, USA) in order to classify genes based on their biological functions and disclose the networks connecting specific genes. IPA uses a variety of computational algorithms to identify and establish cellular networks that statistically fit the input gene list and the molecules present in the Ingenuity Knowledge database. Significant interaction networks were generated by IPA for genes found with likelihood higher than by random chance. Using a 99% confidence level, IPA network scores ≥2 (reflecting the negative logarithm of P values <0.01) were considered significant. The score is used to rank networks according to their relevance to the genes in the input dataset. Canonical pathways analysis identified the pathways most significant for the input data set. The significance of the association between the data set and the canonical pathway was determined based on two parameters: (1) a ratio of the number of genes from the data set that map to the pathway divided by the total number of genes that map to the canonical pathway; and (2) a P value calculated using Fischer’s exact test, determining the probability that the association between the genes in the data set and the canonical pathway is due to chance alone.

**Real-time PCR analysis**

To validate microarray data, quantitative real-time PCR (qRT-PCR) was performed on the same samples used for microarrays experiments and on additional samples obtained under the same experimental conditions. Primer designs were based on the entire coding region for each gene.. One μg of total RNA was used for cDNA synthesis. High-Capacity cDNA Reverse Transcription Kit (Applied Biosystems, Foster City, CA, USA) was used to reverse transcribe RNA to cDNA. The reaction was carried out on a GeneAmp PCR System 9700 (Applied Biosystems) under the following conditions: 10 min at 25 °C, 120 min at 37 °C and 5 min at 85 °C. For each reverse transcription, no-template as well as no-RT controls were additionally included. Quantitative RT-PCR analyses were performed with the ABI PRISM 7000 Sequence Detection System instrument and software (Applied Biosystems). All reactions were performed in a total volume of 25 μL containing 50 ng of cDNA, 0.3 pmol/L of a primer pair and 12.5 μL of the 2x SYBR Green PCR master mix under the following conditions: 2 min at 50 °C, 10 min at 95 °C and 40 cycles of 15 s at 95 °C and 1 min at 60 °C. Reactions were carried out in triplicate on 3 independent sets of RNA. Negative controls (no RNA added) were processed under the same conditions as experimental samples. The critical threshold (CT) value for each target gene was determined using the software provided by the manufacturer and normalized to the expression level of 18S or glyceraldehyde-3-phosphate dehydrogenase (GADPH) used as endogenous controls to normalize target gene expression and to correct for experimental variation.

**Knockdown of CD47 and CARD11 by small interfering RNA(siRNA)**

Gene knockdown experiments were performed by transient transfection exposing HUVEC to a pool of pre-designed siRNA (Qiagen) against CD47 (FlexiTube siRNA: Hs_CD47 _6, 7, 8), CARD11 (FlexiTube siRNA: Hs_CAR11 _1, 4, 7) or to a scrambled sequences (AllStar Negative Control siRNA, 1027281, Qiagen) using DharmaFECT Transfection Reagent (Dharmacon, CO, USA) according to the manufacturer’s protocol. Briefly, HUVECs were plated in 6-well plate the day before transfection at 35.000 cells/cm^2^, in complete medium without antibiotics. Shortly before transfection, culture medium was removed and replaced with 1600 µL of fresh complete medium without antibiotics and returned to the normal growth conditions. To generate transfection complexes, 30 pmol of each siRNA (90 pmol total) or 90 pmol of scrambled sequences in M199 without additives (total 400 µl) were incubated with 3 µL of transfection reagent for 20 minutes at room temperature. The complexes were then added drop-wise to the cells. After 24 h medium was changed with complete medium plus antibiotics. After further 48 h some monolayers were stimulated with 5 ng/mL IL-1β for three hours before total RNA harvesting. The knockdown efficiency was calculated by qRT-PCR using specific primers to CD47 and CARD11. The effects of CD47 and CARD11 knockdown on the expression profile of several genes were analyzed of by qRT-PCR.

**Protein extraction and Western blotting**

In order to verify whether the identified changes in gene expressions translate in modulation of the corresponding proteins HUVECs were preincubated with 50 µmol/L DHA for 0-48 h, followed by stimulation with 5 ng/mL IL-1β for additional 0-24 h. After this time cells were lysed in mammalian cell lysis buffer (Sigma-Aldrich, St. Louis, MO, USA), containing protease and phosphatase inhibitors. Samples were then centrifuged for 10 min at 12000 x g and protein concentration of supernatants determined using a Bio-Rad protein assay kit (Bio-Rad Laboratories, Hercules, CA, USA). Equal amounts of proteins were subjected to electrophoresis in 10% precast sodium dodecyl sulfate-polyacrylamide gel (SDS-PAGE) (Bio-Rad Laboratory). The resolved proteins were transferred to a nitrocellulose membrane (Amersham Biosciences, Cardiff, UK) and, after saturation of nonspecific binding sites, incubated overnight with specific primary antibodies against PDE5α, CD47, TGF-β2, CARD11, CYP4F2 (all from Santa Cruz Biotechnology, Santa Cruz, CA, USA). After incubation with the primary antibody, blots were incubated for 1 h with the pertinent secondary antibody consisting of an anti-rabbit or an anti-goat polyclonal IgG coupled with horseradish peroxidase (Santa Cruz Biotechnology). Protein bands were visualized by an enhanced chemiluminescence kit (Bio-Rad Laboratories ) according to manufacturer’s instructions. Blots were scanned with an Agfa Arcus II scanner (Mortsel, Belgium), and band densities quantified with the aid of the ImageJ 1.47v software.

**REFERENCES**

1. Carluccio MA, Massaro M, Bonfrate C, Siculella L, Maffia M, Nicolardi G, et al. Oleic acid inhibits endothelial activation - A direct vascular antiatherogenic mechanism of a nutritional component in the Mediterranean diet. Arteriosclerosis Thrombosis and Vascular Biology. 1999; 19: 220-228.

2. Buonomo T, Carraresi L, Rossini M, Martinelli R. Involvement of aryl hydrocarbon receptor signaling in the development of small cell lung cancer induced by HPV E6/E7 oncoproteins. J Transl Med. 2011; 9: 2.
