# Supplementary figures and images for: Transcriptome-based identification of new anti-anti-inflammatory and vasodilating properties of the n-3 fatty acid docosahexaenoic acid in vascular endothelial cell under proinflammatory conditions
Source: PLoS One. 2015 Jun 26;10(6):e0129652. doi: 10.1371/journal.pone.0129652 (PMC4482638; doi:10.1371/journal.pone.0129652)

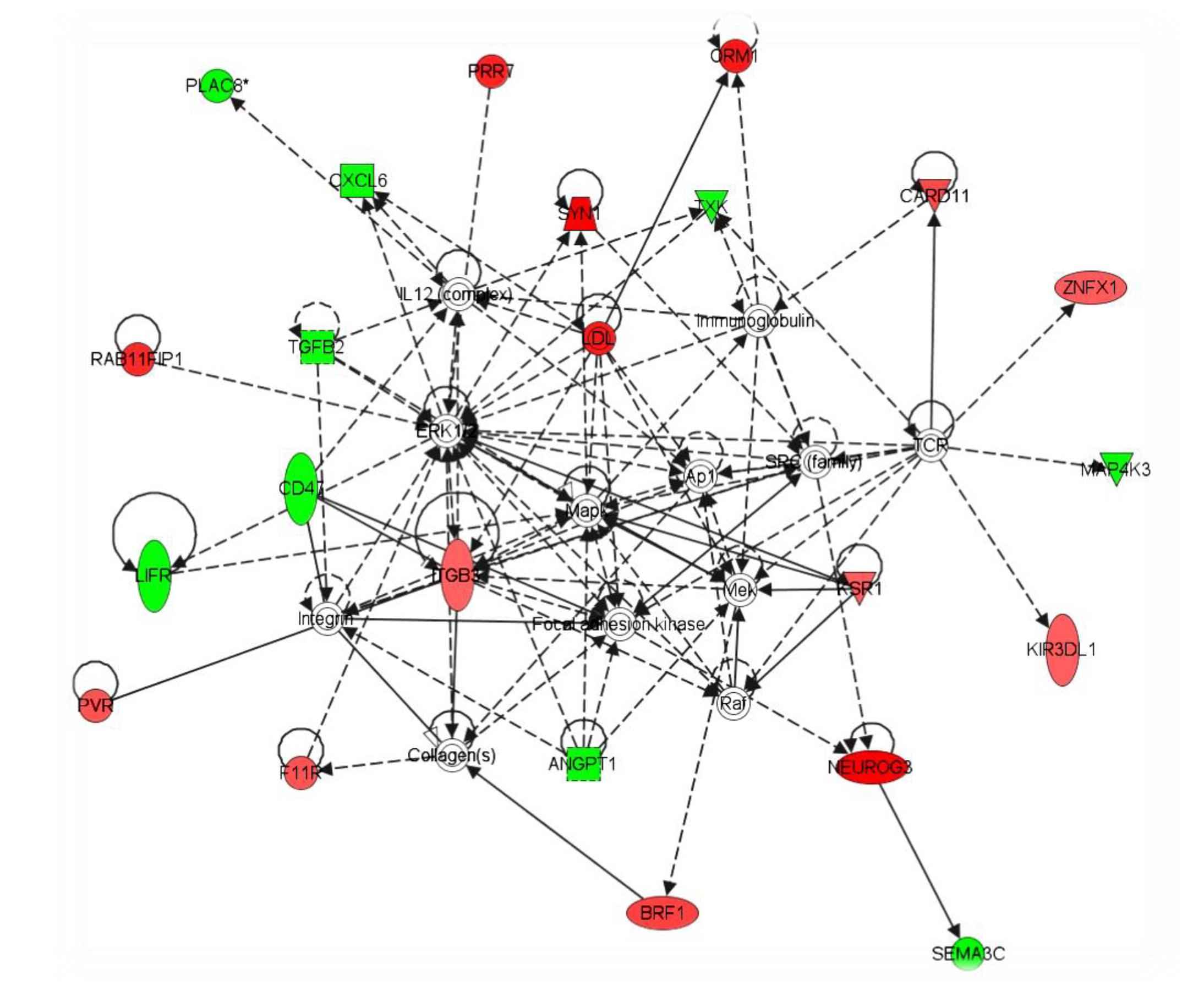

Supplement: S1 Fig — Networks of genes were algorithmically generated with the IPA software based on their connectivity and assigned a score. The intensity of the node color indicates the degree of up- (red) or down-(green) regulation. A continuous line means a direct relationship between the two genes, whereas a discontinuous line indicates an indirect association. The network depicted includes 22 focus molecules. (TIF) [file pone.0129652.s003.tif]

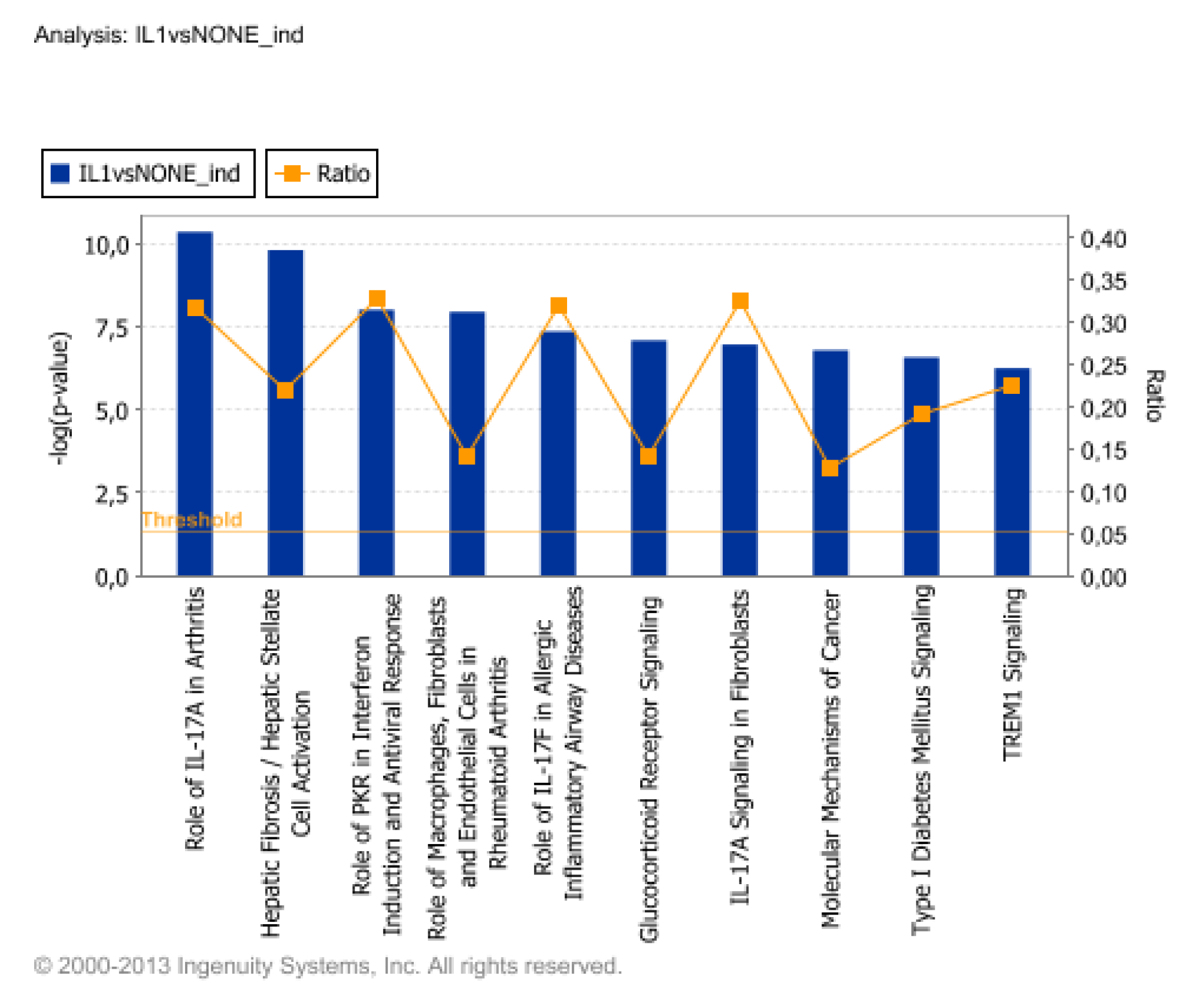

Supplement: S2 Fig — For the functional categorization of genes, Fischer’s exact test was used to calculate a P value (shown as bars) indicating the probability that each biological function assigned to the network is due to chance alone. The ratio (shown as squares) represents the number of differentially expressed genes in a given pathway divided by total number of genes that make up that canonical pathway (TIF) [file pone.0129652.s004.tif]

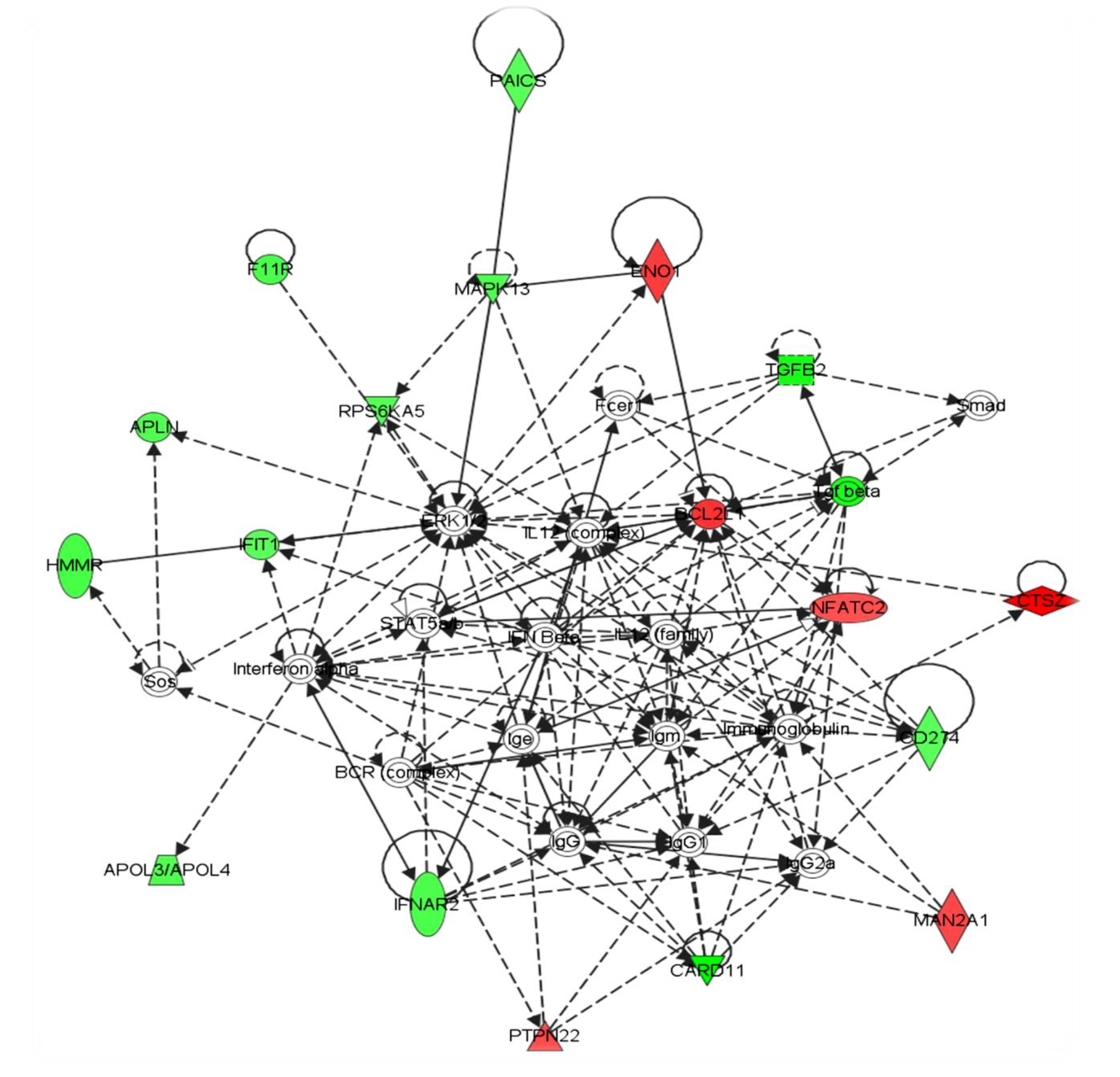

Supplement: S3 Fig — Networks of genes were algorithmically generated with the IPA software based on their connectivity and assigned a score. The intensity of the node color indicates the degree of up- (red) or down-(green) regulation. A continuous line means a direct relationship between the two genes, whereas a discontinuous line indicates an indirect association. The network depicted includes 18 focus molecules. (TIF) [file pone.0129652.s005.tif]

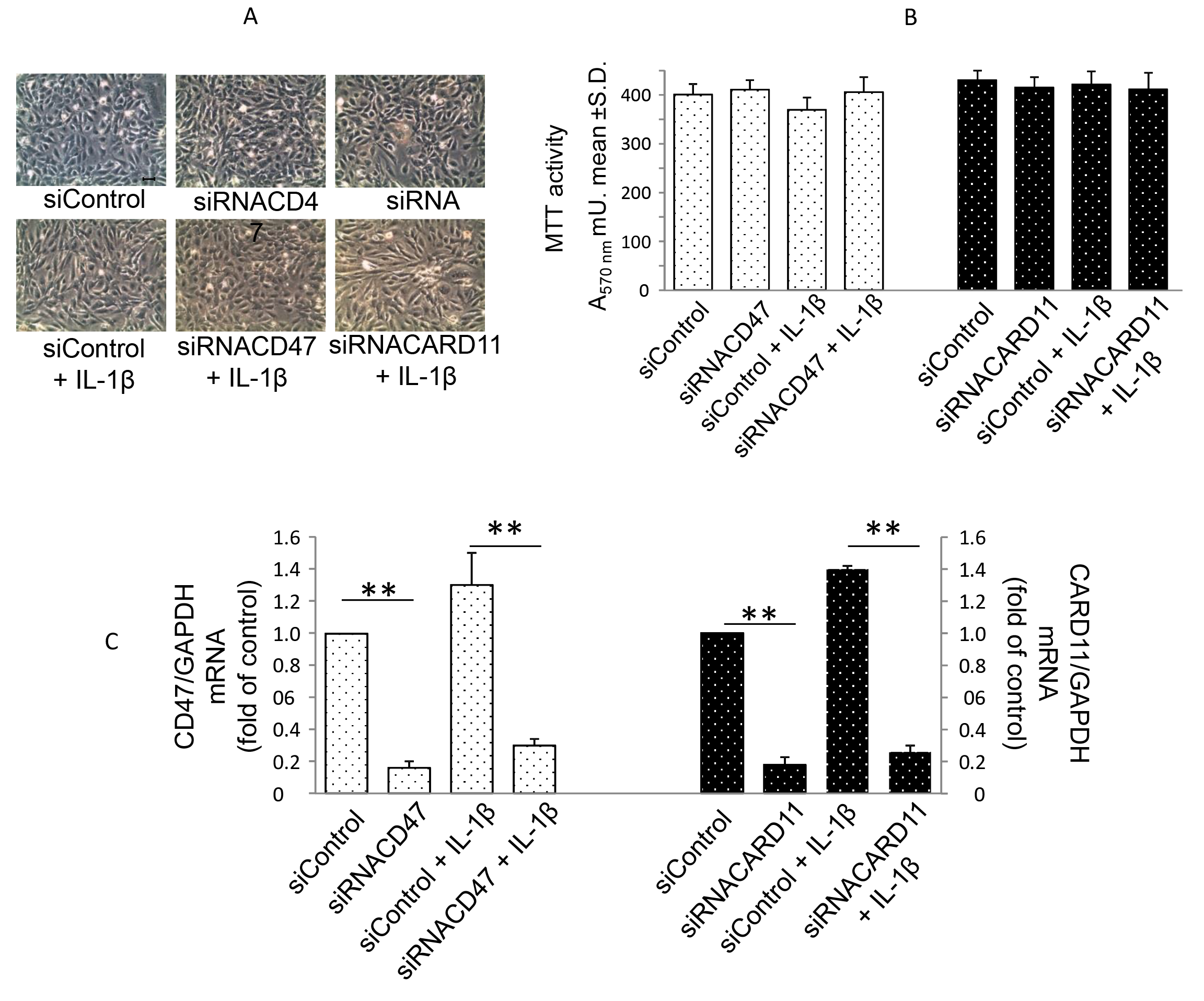

Supplement: S4 Fig — HUVECs were transfected with non-silencing (siControl), CD47 or CARD11 siRNA for 72 before IL-1β stimulation. (A) Phase contrast images of the endothelial monolayers after silencing of CD47 and CAR11. Bars, 20 μm. (B) Effect of Cd47 and CARD11 silencing on endothelial vitality. After gene silencing and IL-1β stimulation MTT assay was performed. In no culture condition tested were highlighted evidence of toxicity by gene silencing. Absorbance data are expressed as milliunits (mU), mean ± standard deviation (S.D.) (n = 16). (C) HUVEC were transfected with CD47 siRNA, CARD11 siRNA or non-silencing siRNA (siControl) for 72 h before 3 h stimulation with 5 ng/mL IL-1β. CD47 and CARD11 mRNA expression levels were analyzed by qRT-PCR. Data are expressed as fold induction over unstimulated siControl and derive from three independent experiments performed in duplicate. **<P<0.01 between groups joined by the horizontal lines. (TIF) [file pone.0129652.s006.tif]

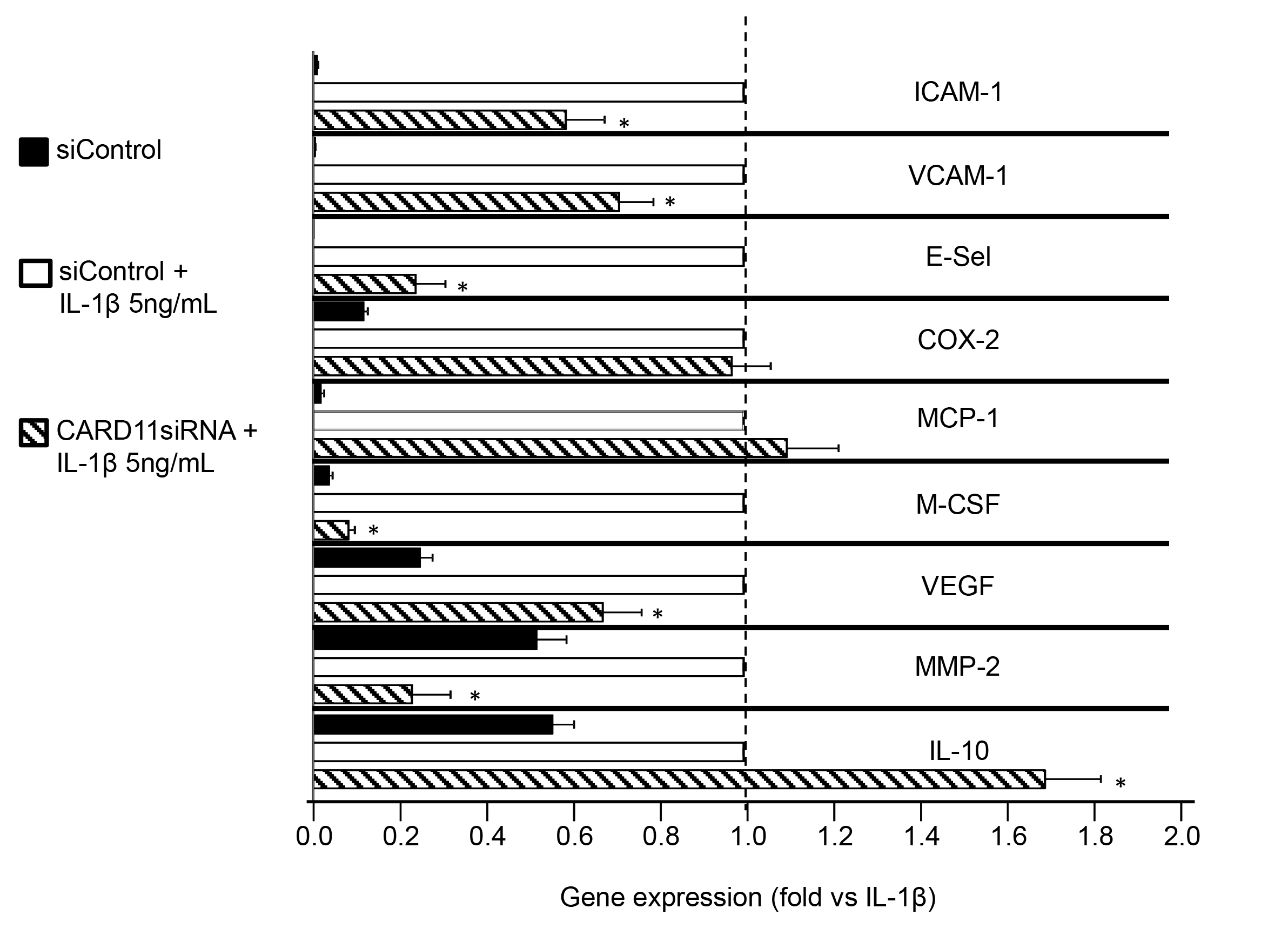

Supplement: S5 Fig — HUVEC were transfected with CARD11 siRNA or nonsilencing siRNA (siControl) for 72 hours. After 3 h stimulation with 5ng/mL IL-1β total RNA was isolated and gene expression profile of the indicated genes assessed by qRT-PCR. Data are presented as fold induction over siControl + IL-1β and derive from two independent experiments performed in duplicate. *P<0.01 vs siControl + IL-1β. (TIF) [file pone.0129652.s007.tif]

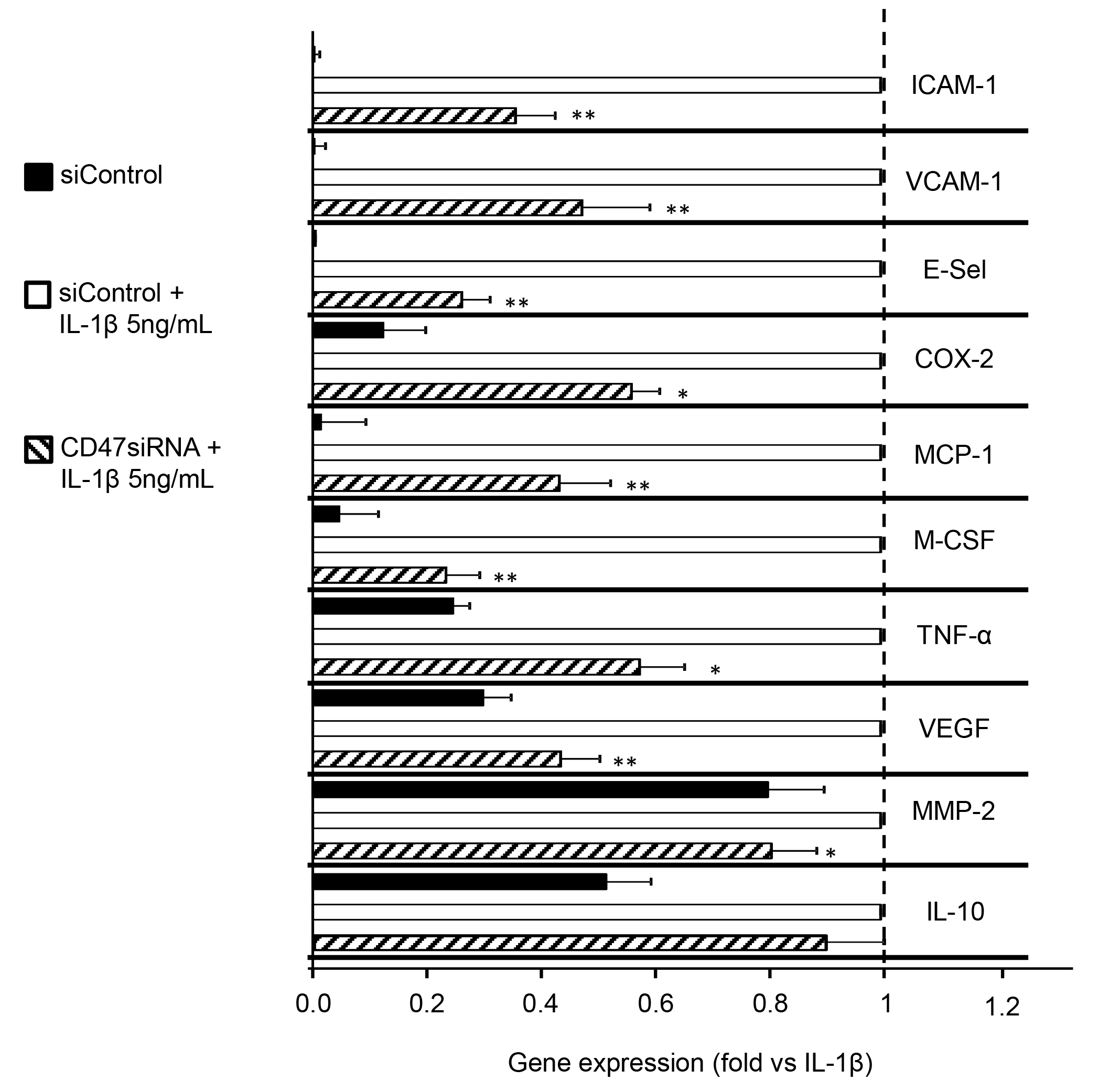

Supplement: S6 Fig — HUVEC were transfected with CD47 siRNA or nonsilencing siRNA (siControl) for 72 hours. After 3 h stimulation with 5 ng/mL IL-1β total RNA was isolated and gene expression profiles of the indicated genes assessed by qRT-PCR. Data are presented as fold induction over siControl + IL-1β and derive from two independent experiments performed in duplicate. *P<0.05 vs siControl + IL-1β; **P<0.0 vs siControl + IL-1β. (TIF) [file pone.0129652.s008.tif]

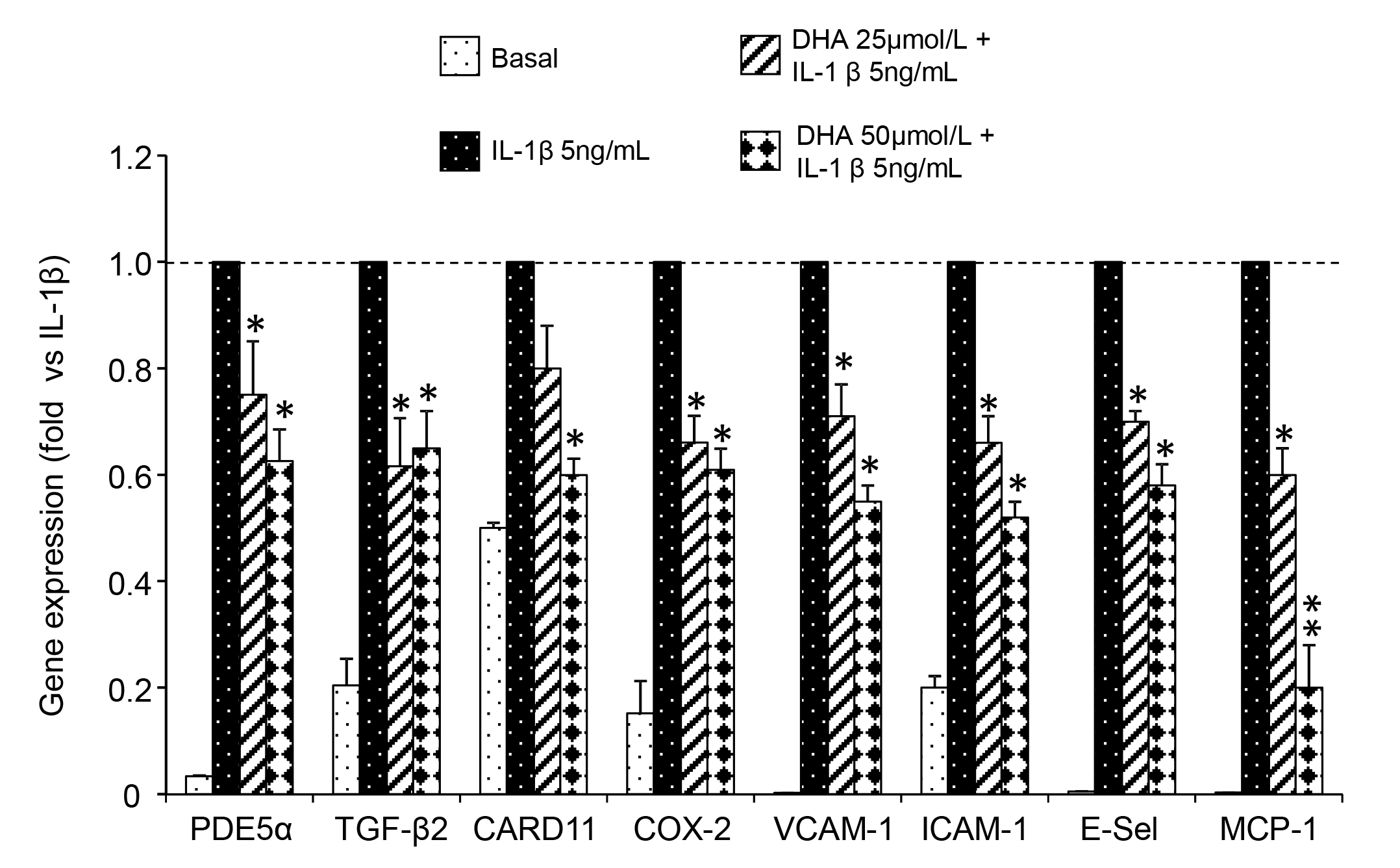

Supplement: S7 Fig — HUVECs were treated with/without DHA for 48 h and then stimulated with IL-1β. After 3 h mRNA level of various genes was assayed by qRT-PCR. Data are expressed as fold induction over unstimulated control and derive from three independent experiments performed in duplicate. *P<0.05 vs IL-1β alone; **P<0.01 vs IL-1β alone. PDE5α, phosphodiesterase 5α; TGF-β2, transforming growth factor-β2; CARD11, caspase recruitment domain-11; COX-2, cyclooxygenase-2; VCAM-1, vascular cell adhesion molecule-1; ICAM-1, intercellular adhesion molecule-1; E-Sel, E-selectin; MCP-1, monocyte chemoattractant protein-1. (TIF) [file pone.0129652.s009.tif]
